# Decoding the Molecular Landscape of Prepubertal Oocyte Maturation: GTPBP4 as a Key Driver of In Vitro Developmental Competence

**DOI:** 10.1111/cpr.70017

**Published:** 2025-02-28

**Authors:** Jianpeng Qin, Yaozong Wei, Ao Ning, Wenqi Hu, Pengcheng Wan, Beijia Cao, Bo Pan, Tianyi Lv, Kunlin Du, Xueling Yao, Shuqi Zou, Xiangyi Chen, Shengqin Zang, Jiangfeng Ye, Guozhi Yu, Qiuxia Liang, Liuhong Shen, Lin Zhang, Xiang Chen, Keren Cheng, Li Meng, Guangbin Zhou

**Affiliations:** ^1^ State Key Laboratory of Swine and Poultry Breeding Industry, Key Laboratory of Livestock and Poultry Multiomics, Ministry of Agriculture and Rural Affairs, Farm Animal Genetic Resources Exploration and Innovation Key Laboratory of Sichuan Province, College of Animal Science and Technology Sichuan Agricultural University Sichuan Chengdu China; ^2^ California Institute of Technology Pasadena California USA; ^3^ State Key Laboratory of Sheep Genetic Improvement and Healthy Breeding, Institute of Animal Husbandry and Veterinary Sciences Xinjiang Academy of Agricultural and Reclamation Sciences Shihezi Xinjiang China; ^4^ College of Life Science Sichuan Agricultural University Ya'an Sichuan China; ^5^ The Key Laboratory of Animal Disease and Human Health of Sichuan Province, The Medical Research Center for Cow Disease, College of Veterinary Medicine Sichuan Agricultural University Chengdu Sichuan China; ^6^ Chengdu Xiling Snow Agricultural Development Co., LTD Chengdu Sichuan China; ^7^ College of Animal Science Guizhou University Guiyang Guizhou China; ^8^ Department of Obstetrics and Gynecology, Center for Reproductive Medicine, the Fourth Affiliated Hospital of School of Medicine and International School of Medicine, International Institutes of Medicine Zhejiang University Yiwu Zhejiang China; ^9^ California IVF Fertility Center Sacramento USA

**Keywords:** adult goat, GTPBP4, oocyte maturation, prepubertal goat, transcriptome, translatome

## Abstract

The intricate mechanisms driving oocyte maturation remain only partially understood, especially within the domains of domestic animal reproduction and translational medicine. In the case of prepubertal girls, the clinical challenge is especially pronounced, as ovarian tissue cryopreservation‐though promising‐remains an experimental technique necessitating rigorous scientific validation to guarantee the developmental potential of preserved materials and facilitate broader clinical adoption. To address these knowledge gaps, while considering the ethical implications, we applied transcriptome and translatome sequencing to comprehensively profile the transcriptional and translational dynamics of oocyte maturation in adult and prepubertal goats. Our analyses uncovered a sequential transition in gene expression regulation, shifting from cytoplasmic processes to chromosome segregation during the maturation process. Comparative profiling between adult and prepubertal goat oocytes revealed critical regulatory factors essential for prepubertal oocyte maturation. These include genes involved in organelle function (*GTPBP4* and *TOMM7*), spindle organisation (*CKS2*, *CCP110*, *CKAP5* and *ESCO1*) and chromosome segregation (*CENPE*, *CENPF*, *CENPN* and *SGO2*). Functional validation through in vitro maturation experiments demonstrated that GTPBP4 significantly enhances the developmental competence of prepubertal goat oocytes. This enhancement occurs through mechanisms that promote cell cycle progression, organelle maturation and mRNA translation. These findings provide a detailed map of the molecular events underpinning goat oocyte maturation and offer new perspectives on the developmental strategies required for oocyte competence in prepubertal females. Translating these insights to humans, this research highlights potential fertility preservation strategies for prepubertal girls, such as ovarian tissue cryopreservation and transplantation, in vitro follicle culture, meiotic maturation and artificial ovary technologies. Moreover, the identified mechanisms have significant implications for improving reproductive efficiency in domestic animal breeding, bridging basic research and applied science.

## Graphical abstract

Transcriptional and translational characteristics during goat oocyte maturation and identification of key factors, including GTPBP4, for prepubertal goat oocyte maturation.

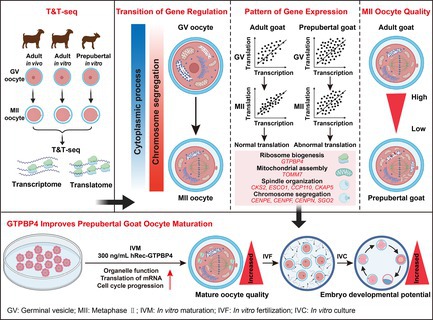

## 1 Introduction

Oocyte maturation is pivotal for successful fertilisation and early embryonic development [1], underpinning both clinical and agricultural reproductive advancements. In animal husbandry, the in vitro maturation (IVM) of oocytes facilitates embryo cloning, in vitro fertilisation (IVF) embryo production, and fundamental investigations into embryogenesis across species such as porcine [2], bovine [3, 4], sheep [5] and goat [6]. Notably, juvenile females ovaries‐unlike their mature counterparts‐exhibit heightened hormonal sensitivity and reduced follicular atresia, allowing the acquisition of a substantial pool of oocytes [7, 8]. Yet, the journey towards optimising juvenile in vitro embryo transfer (JIVET) encounters a formidable obstacle: the inherently low developmental competence of oocytes derived from juvenile females [9, 10, 11], which restricts the broader application of these techniques. Clinically, prepubertal girls face analogous hurdles. While ovarian tissue cryopreservation holds immense promise as a fertility preservation strategy [12], it remains an experimental approach requiring meticulous validation to unlock its full potential and ensure clinical reliability.

Concurrently, advances in paediatric oncology have significantly enhanced survival rates, with over 80% of children and adolescents achieving five‐year survival [13]. However, the life‐saving regimens of chemotherapy and radiation often precipitate premature gonadal insufficiency, jeopardising fertility and hormonal balance [14]. Fertility preservation, therefore, has emerged as a pressing concern for clinicians and patients alike. While standard assisted reproductive technologies (ARTs) offer viable solutions for pubertal patients, these approaches often necessitate hormonal stimulation, which may clash with ongoing medical treatments. For prepubertal girls, however, mature oocyte cryopreservation remains unfeasible due to the immaturity of the hypothalamic–pituitary‐gonadal axis, rendering ovarian stimulation ineffective and retrieved oocytes needing to mature in vitro [15]. Therefore, revealing the molecular mechanism of juvenile females oocyte maturation has important reference significance for fertility preservation in prepubertal girls.

The intricacies of oocyte maturation are governed by granulosa cells, which sustain meiotic prophase arrest via cyclic GMP (cGMP) signalling. Luteinizing hormone (LH) triggers a cascade that diminishes cGMP levels, prompting meiotic resumption [16]. In vitro, the absence of mural granulosa cells necessitates compensatory measures, such as elevated FSH and LH levels, to emulate physiological conditions [17]. However, these interventions can lead to premature luteinizing hormone receptor expression in cumulus cells, accelerating oocyte maturation and deviating from the natural process. Bridging these discrepancies is vital for enhancing oocyte quality in both prepubertal girls and juvenile livestock.

Among the myriad steps of JIVET, IVM of oocytes is paramount, as it is during this stage that oocytes acquire the capacity for fertilisation and early embryonic development [18]. The goat, a versatile experimental model, offers a pathway to address these knowledge gaps while navigating ethical considerations. Despite considerable progress, the molecular mechanisms underlying IVM in juvenile animals and prepubertal girls remain elusive [9, 19]. Therefore, research of IVM of prepubertal goat oocytes can provide important theoretical guidance for the preservation of fertility in prepubertal girls, thus avoiding the ethical limitations of directly using prepubertal girls' ovaries. Emerging technologies, such as single‐cell RNA sequencing, have shed light on mRNA dynamics during oocyte maturation across various species [20, 21, 22]. Yet, the tenuous correlation between mRNA and protein levels underscores the necessity of exploring translational regulation to decipher the functional expression of genes during this process [23].

Innovative methodologies like transcriptome and translatome sequencing (T&T‐seq), capable of simultaneous transcriptome and translatome profiling in as few as 1–10 oocytes, are now unveiling the intricate interplay of transcription and translation during IVM [24]. This cutting‐edge approach has illuminated translation landscapes in aged human and mouse oocytes [25] and holds transformative potential for studying prepubertal girls and juvenile livestock. By employing T&T‐seq, this study delineated transcriptional‐translational landscapes in adult and prepubertal goat oocytes, revealing an uncoupling of these processes. The findings spotlight the pivotal role of functional protein regulation, particularly GTPBP4, in orchestrating cytoplasmic organelle organisation and mRNA translation. Collectively, these insights provide a crucial foundation for addressing reproductive challenges in livestock and advancing the understanding of oocyte maturation in both clinical and agricultural domains.

## 2 Materials and Methods

### 2.1 Animals and Ethics Statement

All experimental protocols adhered strictly to the guidelines of the Animal Ethics and Welfare Committee (AEWC) of Sichuan Agricultural University on January 6, 2016 (Approval code: AEWC2016). Prepubertal goats aged 4–6 weeks and adult goats aged 3–4 years, intended for superovulation, were sourced from Chengdu Xilingxue Agricultural Development Co. Ltd. The animals were housed in well‐ventilated sheds with natural lighting and provided unrestricted access to feed.

### 2.2 Materials

All reagents were purchased from Sigma‐Aldrich (St. Louis, MO, USA) unless specified otherwise.

### 2.3 Goat Superovulation

The superovulation protocol for adult goats was adapted from Yuan et al. [26]. On Day 0, a controlled internal drug release (CIDR) device (InterAg, New Zealand) was inserted vaginally, coupled with a 1.5 mL vitamin ADE injection. From Day 13 to Day 15 post‐CIDR insertion, follicle‐stimulating hormone (FSH) injections (90–110 IU, Ningbo Sansheng Pharmaceutical, China) were administered bi‐daily at 12‐h intervals. Prostaglandin (1 mL, Ningbo Sansheng Pharmaceutical, China) was injected during the final FSH dose, coinciding with CIDR removal. LH (100 IU, Ningbo Sansheng Pharmaceutical, China) was administered 38 h post‐CIDR removal to induce synchronised ovulation (Figure S1A).

For prepubertal goat, the protocol outlined by Liu et al. [27] was employed. A total of six FSH injections (45 IU each) were administered every 12 h, with an additional injection of 400 IU pregnant mare serum gonadotropin (PMSG, Ningbo Sansheng Pharmaceutical, China) during the first FSH dose (Figure S1C).

### 2.4 Collection of Goat Oocytes

For adult goats, germinal vesicle (GV) oocytes were retrieved in vivo following Yuan et al. [26]. At 12 h after the final FSH dose, ovaries were taken out via mid‐ventral laparotomy. Follicles (over 2 mm diameter) were aspirated by an 18 G syringe. The collected cumulus‐oocyte complexes (COCs) were cultured in M199 medium supplemented with 5% FBS (10,099,141, Gibco), 0.42 g/L NaHCO_3_, 4.766 g/L HEPES, 0.05 g/L penicillin and 0.065 g/L streptomycin (Figure S1A).

GV oocytes from slaughterhouse‐sourced adult goat ovaries were processed similarly but transported in 37°C saline and cultured within 2 h of collection (Figure S1B). For prepubertal goats, GV COCs were collected 12 h after the last FSH injection (Figure S1C).

In vivo‐derived metaphase II (MII) oocytes were obtained via oviduct flushing 64 h post‐CIDR removal as per Li et al. [28]. In vitro‐derived MII oocytes from adult and prepubertal goats were matured in M199 medium (11,150,059, Gibco), supplemented with 20% estrous goat serum, 0.33 mmol/L sodium pyruvate, 500 IU/L FSH (F8470, Solarbio), 500 IU/L LH (L8040, Solarbio), 1 mg/L estradiol, 50 μmol/L L‐cystine and 25 mg/L gentamicin. Following 24 h of culture at 38.5°C under 5% CO_2_, COCs were transferred to 0.2% hyaluronidase medium to denude cumulus cells and select matured oocytes for subsequent experiments (Figure S1B,C).

### 2.5 T&T‐Seq for Goat Oocytes

The T&T‐seq protocol was based on Hu et al. [24]. Zona pellucida removal was achieved by immersing 10 GV or MII oocytes in acid Tyrode's solution for 30 s, followed by PBS washes. Lysates from oocytes were prepared in 10 μL ice‐cold lysis buffer (N712, Vazyme) and divided for transcriptome and translatome analyses. Translatome mRNA was isolated using RiboLace Beads (RL001, Immagina), purified and reverse‐transcribed via the Single Cell Full Length mRNA‐Amplification Kit (N712, Vazyme). Libraries were constructed with the TruePrep DNA Library Prep Kit V2 (TD502/503, Vazyme) and sequenced on the Illumina NovaSeq 6000 platform.

### 2.6 T&T‐Seq Data Processing

The raw sequencing data of fastq format were demultiplexed using bcl2fastq2 tool (v.2.20.0.422). Adapter sequences and barcodes were eliminated with Trimmomatic tool (v.0.32). The cleaned fastq data were aligned to the human (GRCh38), mouse (mm39) and goat (ARS1) reference genomes using Rsubread. Gene‐level raw counts were obtained using featureCounts. To ensure robust downstream analysis, lowly expressed genes were filtered out using the filterByExpr function in edgeR with default settings. The raw counts were normalised to the library size and only genes with counts per million (CPM) greater than 10 were retained. The analysis of differential expression was performed by the edgeR (v.3.36.0) and DESeq2. Reads per kilobase per million (RPKM) values were calculated by edgeR software, with the gene lengths sourced from the UCSC Table Browser. Data analyses and visualisations were performed using R software (v.4.1.0). The hierarchical clustering of genes and samples was performed using the hclust function in R. The value of gene expression is normalised by Log_2_(CPM + 1). Differentially expressed genes (DEGs) (absolute Log_2_ fold change > 1, *padj* value < 0.01).

### 2.7 Translational Activity Analysis

Translational activity was calculated as the translatome‐to‐transcriptome ratio using DESeq2. Genes with Log_2_ fold change > 1 and *padj* < 0.01 were indicated translationally active, while Log_2_ fold change < −1 with *padj* < 0.01 indicated repression.

### 2.8 Observation of Goat Oocytes Ultrastructure

Goat oocytes were initially fixed in 3% glutaraldehyde overnight at 4°C and subsequently rinsed three times in PBS for 10 min each. Then, the oocytes were fixed with 1% osmium tetroxide (18,456, Ted Pella) for 2.5 h at 25°C, followed by another series of three 10‐min washes in PBS. For dehydration, the oocytes were treated with increasing concentrations of acetone (30%–50%–70%–80%–90%–95%–100%), with each step lasting 15 min. The 100% acetone treatment was repeated three times. Dehydrated oocytes were permeated sequentially in acetone and Epon812 resin at ratios of 1:1 and 1:3 for 1 and 3 h, respectively. The samples were then embedded in pure Epon812 resin and left at 4°C overnight. 90‐nanometer ultrathin sections were prepared using a diamond knife, followed by double‐contrast staining with 5% uranyl acetate (19481, Ted Pella) and 2.5% lead citrate (19312, Ted Pella). The ultrastructure of oocytes was observed and imaged by transmission electron microscope (TEM, JEM‐1400FLASH, Japan).

### 2.9 IVM of Prepubertal Goat Oocytes

Recombinant hRec‐GTPBP4 (P8248, Fine Test) was supplemented to IVM medium at final concentrations of 0, 100, 300 or 500 ng/mL. After 24 h of maturation, COCs were transferred to 0.2% hyaluronidase medium to eliminate cumulus cells. The denuded oocytes were then selected for subsequent experiments. To assess the percentage of different meiotic stages, oocytes were placed on slides with VECTASHIELD mounting medium with DAPI (H1200, Vector). Slides were sealed with glass coverslips and visualised using a confocal microscope (A1R, Nikon). Meiotic nuclear stages were classified based on established criteria [3], as follows: GV stage, presence of an intact germinal vesicle; GVBD stage, absence of the germinal vesicle, with chromatin condensed into clumps; MI stage, chromosomes aligned on the equatorial plate; MII stage, first polar body extrusion and chromosomes aligned on the equatorial plate.

### 2.10 IVF and Embryo Culture

After 24 h of maturation, COCs were transferred to 0.2% hyaluronidase medium to remove cumulus cells. The denuded oocytes were then transferred to droplets of IVF medium (71004, IVF Bioscience). Fresh sperm were prepared by layering them in equal proportions of PureSperm 40/80 (PS80‐100, PS40‐100, Nidacon) and centrifuging at 500 g for 10 min. The resulting sperm pellet was resuspended in 5 mL of HEPES‐buffered synthetic oviduct fluid medium (H‐SOF) containing 2% FBS and 0.01 g/L heparin sodium and centrifuged again at 300*g* for 5 min. After removing the supernatant, sperm concentration was adjusted to 1 × 10^6^ sperm/mL in IVF droplets. After oocytes and sperm were co‐cultured for 20–22 h, the presumed zygotes were washed and transferred to the droplets of embryo in vitro culture (IVC) medium (71,005, IVF Bioscience) covered with mineral oil. Embryo culture was performed in an incubator (APM‐30D, Astec) at 38.5°C with 5% CO₂, 5% O₂ and 90% N₂ in a humidified atmosphere. The blastocyst rate was calculated on Day 7 based on the number of cleaved embryos.

### 2.11 Immunofluorescence

Oocytes were fixed in 4% paraformaldehyde (PFA) for 1 h and permeabilised in PBS containing 1% Triton X‐100 for 30 min at room temperature. Blocking was performed using PBS with 10% donkey serum for 1.5 h at room temperature. Then, oocytes were incubated overnight at 4°C with the following primary antibodies: Rabbit anti‐GTPBP4 (1:100, Proteintech, 13897‐1‐AP) and Rabbit anti‐RPL7A (1:100, Proteintech, 15340‐1‐AP). Following incubation, oocytes were washed three times in a washing buffer (0.01% Triton X‐100 and 0.1% Tween 20 in PBS) for 10 min each. Secondary antibody staining was performed using CoraLite488‐conjugated Donkey Anti‐Rabbit IgG (1:200, Proteintech, SA00013‐6) at 37°C for 1.5 h. After staining, oocytes were washed again in washing buffer three times and mounted on slides using VECTASHIELD mounting medium. Slides were sealed with glass coverslips and imaged under a confocal microscope (A1R, Nikon). Fluorescence intensity measurements were performed on both control and treatment groups using the same immunostaining and imaging parameters. The average fluorescence intensity per unit area in each oocyte was quantified using ImageJ software (v1.48, Bethesda, USA).

### 2.12 Protein Synthesis Assays

Protein synthesis levels in matured prepubertal goat oocytes were evaluated using the Click‐iT Protein Synthesis Assay Kit (C10428, Thermo Fisher Scientific). Oocytes were cultured in an L‐methionine‐free medium with 50 μM L‐homopropargylglycine (HPG) for 1 h at 37°C. Following incubation, oocytes were fixed in 4% PFA for 1 h and permeabilised in PBS with 1% Triton X‐100 for 30 min at room temperature. After permeabilisation, oocytes were incubated in the reaction cocktail provided in the assay kit for 30 min at room temperature, followed by rinsing in the reaction rinse buffer. Oocytes were then mounted onto slides using VECTASHIELD mounting medium, sealed with glass coverslips and imaged under a fluorescence microscope (BX53, Olympus). The fluorescence intensity corresponding to protein synthesis in each oocyte was quantified using ImageJ software (v1.48, Bethesda, USA).

### 2.13 Mitochondrial Distribution Evaluation

To assess mitochondrial distribution, oocytes were cultured in M199 medium supplemented with 2 μM MitoTracker Red (M7512, Thermo Fisher Scientific) for 30 min at 37°C. After incubation, oocytes were washed in PBS with 1% polyvinylpyrrolidone (PVP) three times for 5 min each to remove excess dye. The oocytes were then fixed in 4% PFA for 1 h and mounted onto slides using VECTASHIELD mounting medium. Slides were sealed with glass coverslips and imaged under a confocal microscope (A1R, Nikon). Mitochondrial distribution was categorised based on established criteria [3]: Uniform distribution, homogeneous distribution throughout the cytoplasm; nonuniform distribution, heterogeneous distribution throughout the cytoplasm.

### 2.14 Statistical Analysis

Statistical analyses were performed using SPSS software (Version 20.0, IBM, USA). The Student's *t*‐test and LSD test were employed under the assumptions of equal variance and normal data distribution. Statistical significance was decided as *p* < 0.05, and error bars represent standard deviation (SD). Before statistical analysis, percentage data were arcsine‐transformed to meet the assumptions of parametric tests. Functional enrichment analysis was performed using Gene Ontology (GO) annotations in the DAVID database (https://david.ncifcrf.gov/). Experimental flowcharts were designed using BioRender (https://app.biorender.com/).

## 3 Results

### 3.1 The Transcriptional and Translational Landscapes of Goat Oocytes Show Unique Features Compared With Human and Mouse

**FIGURE 1:**
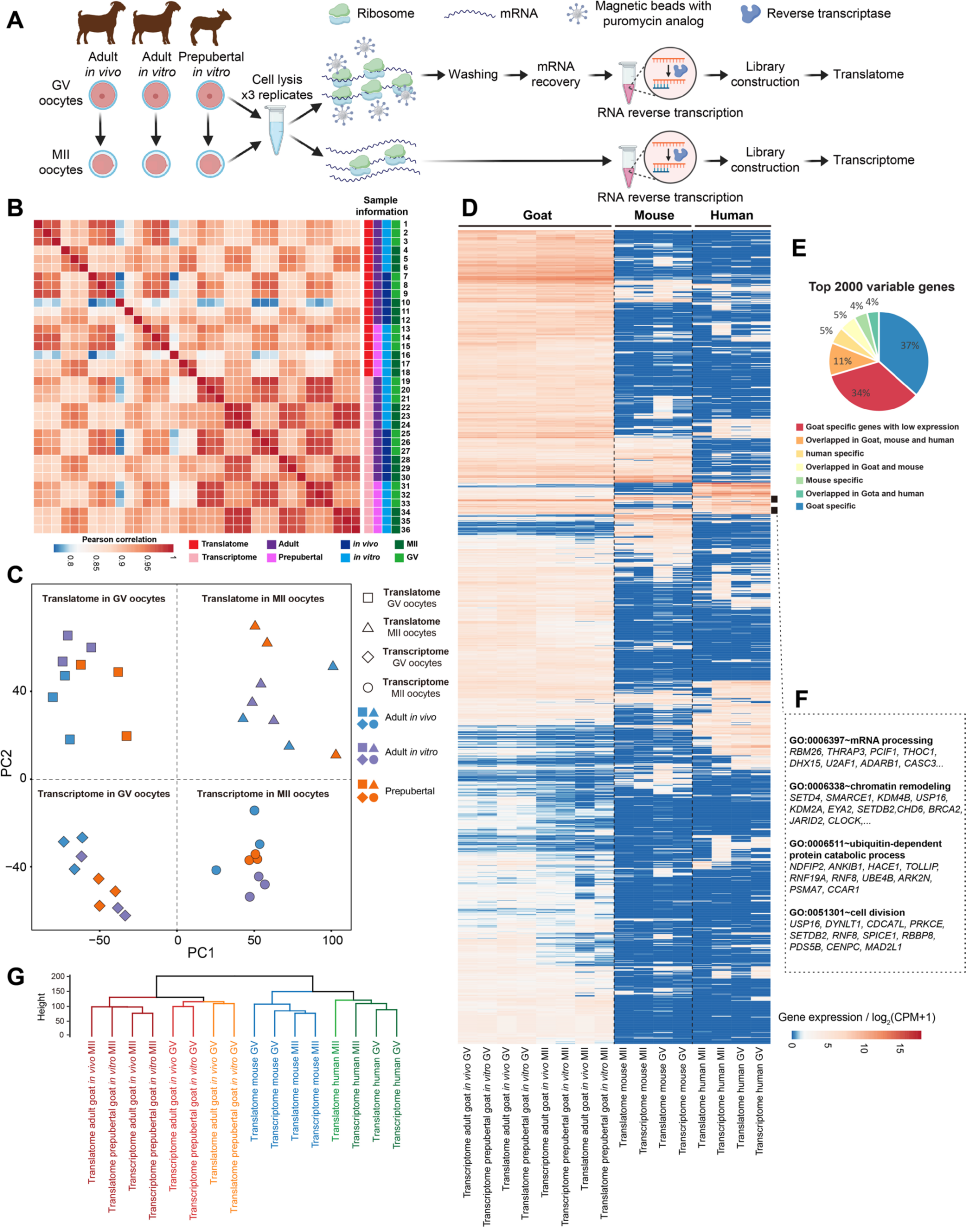
Transcriptome and translatome profiles of goat oocytes generated by T&T‐seq. (A) Experimental flowcharts of T&T‐seq. (B) Correlation coefficient heatmap of the transcriptome and translatome using different goat oocytes. (C) PCA plot of T&T‐seq data from goat oocytes. Red area covers the translatome of GV oocytes, blue area covers the transcriptome of GV oocytes, grey area covers the translatome of MII oocytes and purple area covers the transcriptome of MII oocytes. (D) Expression heatmap of the top 2000 most variable genes from goat, mouse and human oocytes. (E) Pie charts showing the proportion of overlapped and specific genes in goat, mouse and human. (F) Representative GO BP terms enrichment of overlapped genes in goat, mouse and human. (G) Hierarchical clustering of transcriptome and translatome data from goat, mouse and human oocytes.

The translational regulation is widely recognised as a regulatory step in oocyte maturation. However, the scarcity of oocytes and limitations of bulk sequencing techniques have hindered simultaneous profiling of the transcriptome and translatome until recently (Figure 1A). To elucidate the gene expression and translational regulations in goat oocytes during maturation, we conducted concurrent T&T‐seq at both GV and MII stages simultaneously. Each sample of our T&T‐seq yielded between 38 and 65 million high‐quality reads (Figure S2A and Data S1). The correlation coefficients demonstrated a strong similarity between the replicates in terms of the transcriptome and translatome (Figure 1B). To summarise the features of goat oocytes' transcriptome and translatome, we performed principal component analyses (PCA), which clearly separated the transcriptomes and translatomes into distinct parts. Additionally, goat GV oocytes exhibited a developmental trajectory towards MII oocytes (Figure 1C). Next, we aimed to identify the unique features in goat oocytes by comparing our datasets with previously published mouse and human T&T‐seq datasets [24] and discovered that 71% of the top 2000 variable genes were specific to goat (Figure 1D,E). However, there were still 212 genes expressed in oocytes across all three species (Figure 1E), characterised by their involvement in mRNA processing, chromatin remodelling, ubiquitin‐dependent protein catabolic process and cell division. This suggests that these processes were fundamental for oocyte maturation (Figure 1F). Furthermore, we observed that goat oocytes exhibited relatively greater differences from human oocytes than from mouse ones in terms of both transcriptome and translatome profiles (Figure 1G and Figure S2B–I).

### 3.2 Profiling the Synchronised Transcriptome and Translatome of Adult Goat Oocytes

**FIGURE 2:**
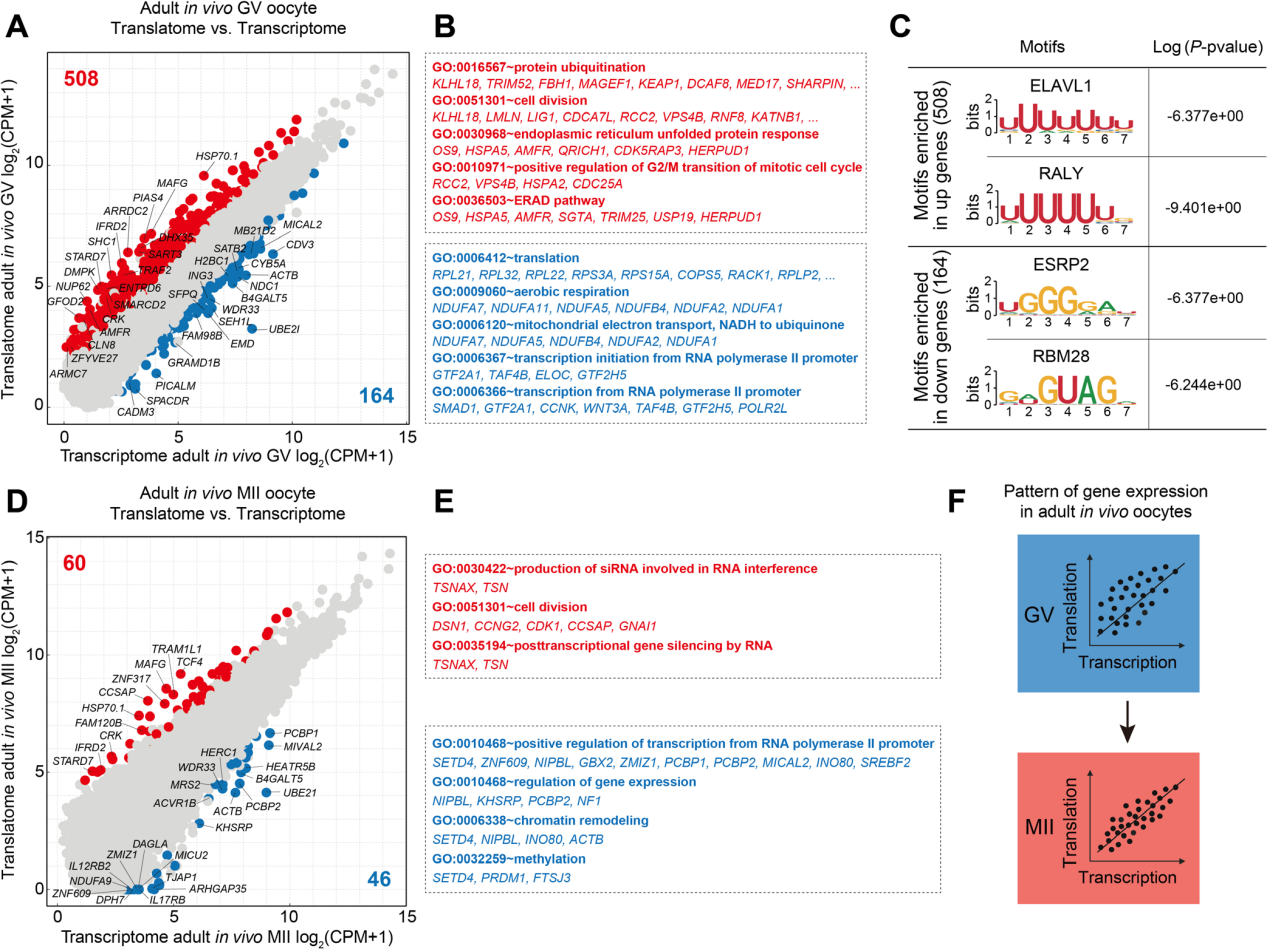
Profiling the transcriptome and translatome of adult goat GV and MII oocytes in vivo. (A) Scatter plots comparing average gene expression values between transcriptional and translational levels from adult goat GV oocytes in vivo. (B) Representative GO BP terms enrichment of translationally active genes (red colour circle) and translationally repressed genes (blue colour circle) showed in Figure 2A, respectively. (C) Putative regulators identified in 3′ UTR motifs of translationally active genes and translationally repressed genes in adult goat GV oocytes. (D) Scatter plots comparing average gene expression values between transcriptional and translational levels from adult goat MII oocytes in vivo. (E) Representative genes and their GO BP enrichments for DEGs in Figure 2D, respectively. (F) Pattern of gene expression in adult goat oocytes.

During late oogenesis, GV oocytes exhibit transcriptional silencing due to chromatin remodelling [29]. Therefore, our aim was to comprehend the pattern of gene expression that distinguishes adult goat GV and MII oocytes. Intriguingly, we have discovered that adult goat GV oocytes exhibited approximately 508 translationally active genes (Figure 2A), characterised by protein ubiquitination, cell division regulation and response to endoplasmic reticulum stress (Figure 2B). Conversely, there were 164 genes in these cells that were translationally repressed and associated with cytoplasmic translation, mitochondrial ATP synthesis coupled with proton transport and aerobic respiration (Figure 2A,B). To identify additional regulatory elements, we conducted motif enrichment analysis using 3′ UTRs of DEGs. Predicted regulators such as *ELAVL1* and *RALY* exhibit preferential binding to genes with translation activity, while *ESRP2* and *RBM2*8 may potentially suppress the translation of certain genes (Figure 2C).

Next, we herein profiled the transcriptome and translatome of adult goat MII oocytes simultaneously. Through pair‐wise comparison, we identified 106 genes that exhibited differential regulation between the transcriptome and translatome, indicating coordinated transcription and translation of genes during profiling (Figure 2D). Notably, genes with higher translating activity were found to be enriched in siRNA production involved in RNA interference, cell division, and posttranscriptional gene silencing by RNA (Figure 2E). Conversely, genes with lower translational activity showed enrichment in positive regulation of transcription from RNA polymerase II promoter (*SETD4*, *ZNF609*, *PCBP1* and *PCBP2*) as well as chromatin remodelling and methylation processes (Figure 2E). Our results demonstrated distinct patterns of transcription and translation in adult goat GV oocytes, but close coupling in MII oocytes (Figure 2F).

### 3.3 Sophisticated Posttranscriptional Regulation of mRNA Revealed by Co‐Profiling Transcriptome and Translatome During Adult Goat Oocyte In Vivo Maturation

**FIGURE 3:**
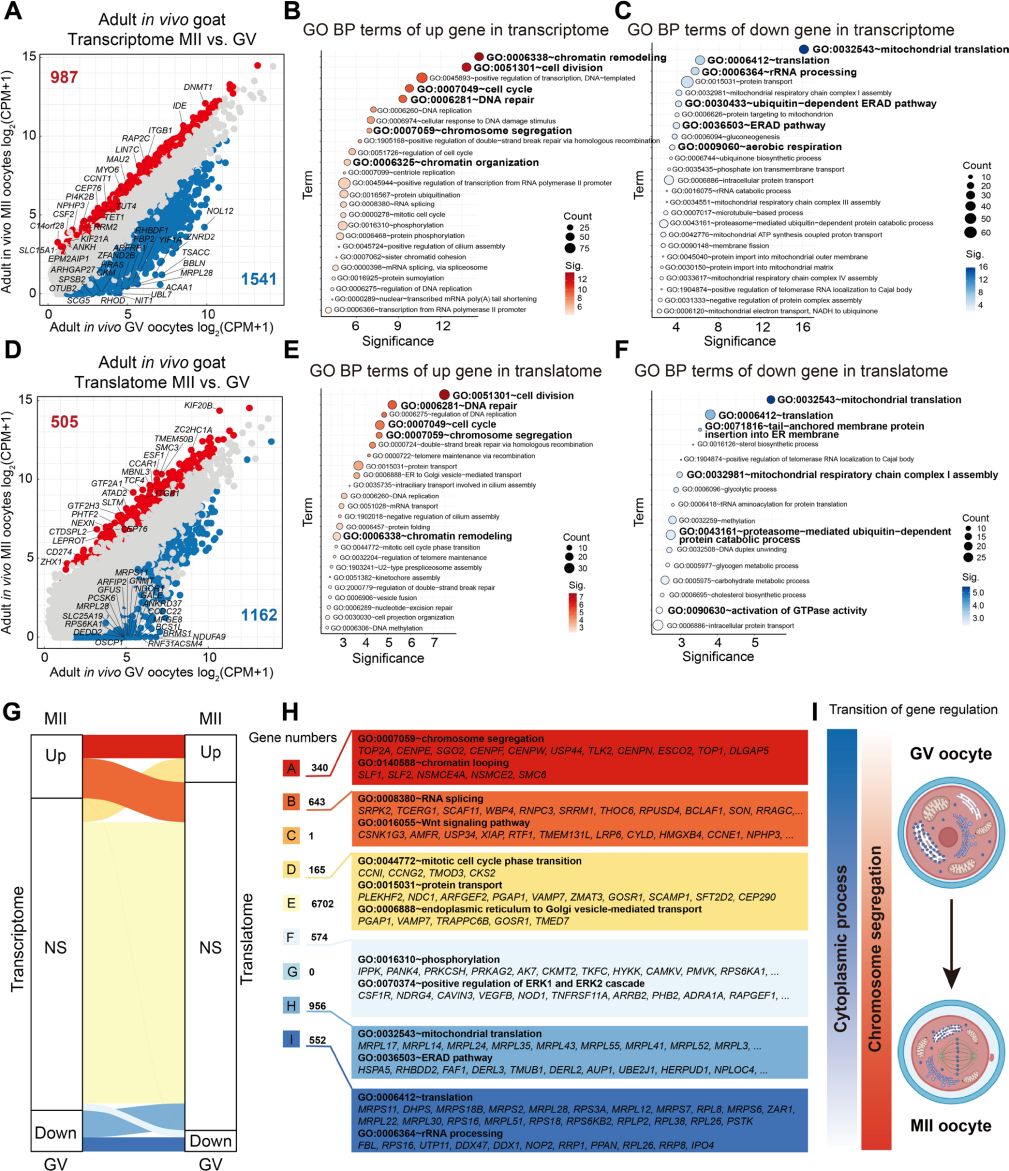
Co‐profiling transcriptome and translatome during in vivo oocyte maturation from adult goat. (A) Scatter plots comparing average gene expression values between GV and MII oocytes in transcriptional levels. (B and C) Representative GO BP terms enrichment of up‐regulated (red colour circle) and down‐regulated (blue colour circle) genes showed in Figure 3A, respectively. (D) Scatter plots comparing average gene expression values between GV and MII oocytes in translational levels. (E and F) Representative GO BP terms enrichment of up‐regulated (red colour circle) and down‐regulated (blue colour cirlce) genes showed in Figure 3D, respectively. (G) Alluvial diagram showing the kinetics of gene expression during maturation of adult goat oocytes in vivo. (H) Representative GO BP terms of different classes of genes showed in Figure 3G, respectively. (I) Transition of gene regulation during maturation of goat oocytes.

We aimed to elucidate the transcriptome and translatome dynamics during in vivo maturation of adult goat oocytes. Comparative analysis of the transcriptomes revealed that 987 genes were up‐regulated, while 1541 genes were down‐regulated (Figure 3A), showing extensive transcriptional transitions characterised by up‐regulation of processes related to chromatin remodelling, cell division, DNA repair, and chromosome segregation, as well as down‐regulation of mitochondrial translation, general translation machinery, ERAD pathway, and rRNA processing during oocyte maturation (Figure 3B,C and Figure S3A,B). Regarding the translatome dynamics during in vivo maturation of adult goat oocytes, we observed an up‐regulation of 505 genes with enriched functions associated with chromatin remodelling, chromosome segregation, DNA repair and cell cycle progression (Figure 3D,E and Figure S3B). Simultaneously, a total of 1162 genes exhibited down‐regulation with functional enrichment in translation regulation, mitochondrial respiratory chain complex I assembly and GTPase activity activation (Figure 3D,F and Figure S3A).

Subsequently, we categorised the genes into eight groups based on their transcription and translation dynamics during oocyte maturation (Figure 3G and Data S2). Notably, only 340 genes exhibited up‐regulation in both transcription and translation, with a particular emphasis on genes associated with chromosome segregation, chromatin looping, and chromatin remodelling (Figure 3G,H). Additionally, 643 genes displayed higher levels of transcription but were retained for translation during this transition phase. These genes were characterised by their involvement in phosphorylation, RNA splicing and the Wnt signalling pathway (Figure 3G,H). Intriguingly, only one gene (*ZNF609*) showed significant repression in translation while being up‐regulated in transcription. *ZNF609*'s transcript forms a covalently closed loop structure through back‐splicing, which regulates the cell cycle transition in cancer cells [30]. Furthermore, 165 genes were actively translated during oocyte maturation, featured in mitotic cell cycle phase transition, protein transport, and endoplasmic reticulum to Golgi vesicle‐mediated transport (Figure 3G,H). We also observed that 552 genes exhibited down‐regulation in both transcription and translation; these genes were involved in translation and rRNA processing (Figure 3G,H). Our results highlighted the sequential transition of gene expression regulation from cytoplasm to chromosome segregation during this process (Figure 3I).

### 3.4 The Transcriptome and Translatome During IVM of Adult Goat Oocytes Were Different From In Vivo

Due to the lack of systematic investigation into the dynamics of the transcriptome and translatome during IVM of adult goat oocytes, we have summarised the concurrent alterations in the transcriptome and translatome during this process. Our findings revealed that gene transcription during IVM leads to the down‐regulation of 1989 genes involved in mitochondrial translation, aerobic respiration, rRNA processing and the ERAD pathway (Figures S4A–C and S5A). Conversely, 969 genes associated with chromatin remodelling, DNA repair, cell division, and chromosome segregation were up‐regulated (Figures S4A–C and S5B). Moreover, mRNA translation dynamics indicated the down‐regulation of 1587 genes, while 972 genes exhibited up‐regulation (Figure S4D). The shift observed in the translatome from processes such as aerobic respiration, translation, rRNA processing and the ERAD pathway towards cell division, chromatin remodelling, chromosome segregation, and DNA repair during IVM was consistent with the dynamics observed at the transcription level (Figures S4E,F and S5A,B). Furthermore, based on gene transcription and translation dynamics during oocyte maturation, we categorised these genes into nine groups (Figure S4G,H and Data S3). The sequential transitions of gene expression regulation were found to be consistent with the maturation process of adult goat oocyte maturation in vivo (Figure S4G,H and Figure 3G,H).

However, as anticipated, we observed that the distinct patterns of transcriptome and translatome between in vivo and IVM of adult goat oocytes (Figure S4I,J). At the transcriptome level, there were 474 genes that exhibited expected high transcription levels but failed to do so in vitro. Conversely, 456 genes displayed unexpectedly high transcription levels during IVM despite not being predicted to do so. Moreover, 774 genes were specifically down‐regulated during IVM but remained unaffected in vivo; conversely, 326 genes should have been down‐regulated but showed no decrease during the process of IVM. At the translatome level, there were 128 genes that should have undergone significant translation but did not exhibit this behaviour under our experimental conditions. Additionally, 595 genes displayed elevated translation levels during IVM despite not being expected to do so. Furthermore, 1035 genes were down‐regulated in vitro but not in vivo; while 610 genes should be down‐regulated but not in vitro. These results indicated that gene transcription and translation dynamics during adult goat oocyte maturation differ between in vivo and in vitro conditions. This discrepancy is likely influenced by suboptimal culture conditions, which fail to replicate the in vivo microenvironment.

### 3.5 Prepubertal Goat Oocytes Show Aberrant Translational Dynamics During IVM

**FIGURE 4:**
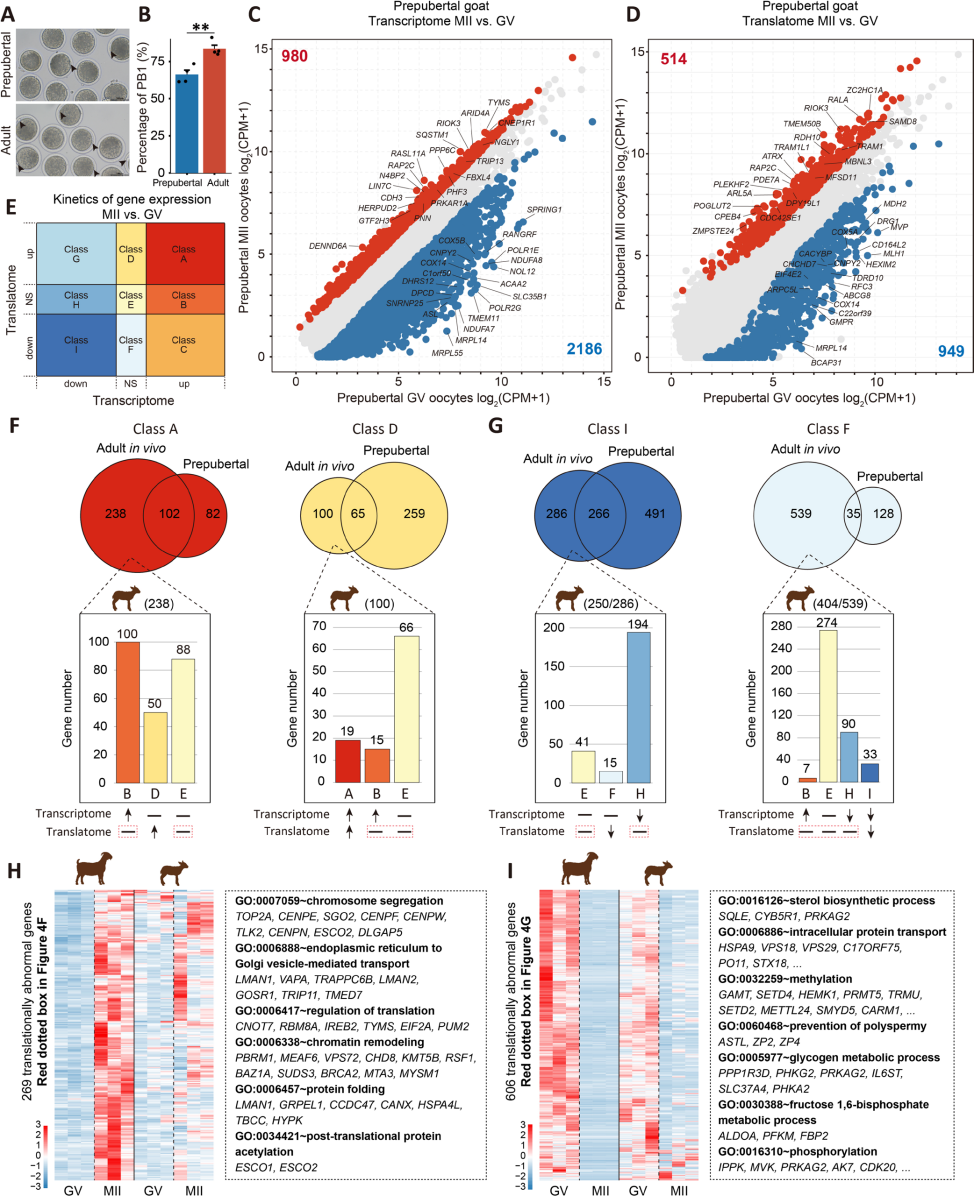
Kinetics of gene transcriptional and translational during in vitro oocyte maturation from prepubertal goat. (A) The images of in vitro matured oocytes from adult and prepubertal goat. Bar = 100 μm. (B) After 24 h in vitro maturation, the percentage of first polar body exclusion. The data were displayed as mean ± SD of four independent experiments. Prepubertal, *n* = 57 oocytes; adult, *n* = 48 oocytes. **, *p* < 0.01. (C and D) Scatter plots comparing average gene expression values between GV and MII oocytes in transcriptional and translational levels, respectively. (E) The kinetics of gene expression during maturation of goat oocytes. (F) Veen plot shows the overlap of class A and class D between adult goat and prepubertal goat. Graph shows the gene transcription and translation dynamics, these genes (class A and class D) were aberrant expression during the IVM process of prepubertal goat oocytes compared to adult goat. (G) Veen plot shows the overlap of class I and class F between adult goat and prepubertal goat. Graph shows the gene transcription and translation dynamics, these genes (class I and class F) were aberrant expression during the IVM process of prepubertal goat oocytes compared to adult goat. (H) The genes exhibited up‐regulation in translatome during adult goat oocytes in vivo maturation but failed to do so prepubertal goat. Representative GO BP terms enrichment of 269 genes showed at right. (I) The genes exhibited down‐regulation in translatome during adult goat oocytes in vivo maturation but failed to do so prepubertal goat. Representative GO BP terms enrichment of 606 genes showed at right.

The abundance of GV oocytes in prepubertal goats enables fast breeding and offers a greater genetic resource compared to adult goats [18, 31]. However, the ratio of prepubertal goat oocytes progressing from the GV stage to the MII stage in vitro culture was found to be lower than that of adult goats (62.29% ± 5.80% vs. 83.49% ± 5.00%) (Figure 4A,B). Therefore, we investigated the dynamic changes in gene transcription and translation during IVM of prepubertal goat oocytes. Interestingly, the transitions of gene transcription and translation from the GV stage to the MII stage in prepubertal goat oocytes were consistent with that observed in adult goats (Figure 3G,H; Figures S6E,F and S4G,H). In terms of gene transcription during IVM, a total of 2186 genes involved in mitochondrial translation, aerobic respiration, and rRNA processing were down‐regulated, while 980 genes associated with chromatin remodelling, DNA repair, and cell division were up‐regulated (Figure 4C; Figures S6A,B and S7A,B). Furthermore, at the level of mRNA translation dynamics, 949 genes showed down‐regulation while 514 genes exhibited up‐regulation (Figure 4D). The shifts observed in translatome patterns align with transcriptome changes involving processes such as aerobic respiration, translation, and rRNA processing towards cell division, cell cycle regulation, and DNA repair (Figures S6C,D and S7A,B). Subsequently, to elucidate the underlying factors contributing to the low IVM rate of prepubertal goat oocytes, we conducted a comparative analysis of gene transcription and translation dynamics between adult goats and prepubertal goat oocytes (Figure S6E,F and Data S4). Considering that proteins are the main executors of life activities and the translatome more effectively reflects protein expression levels than the transcriptome our focus was on comparing gene clusters exhibiting significant changes in translation levels, specifically class A, class D, class F, and class I (Figure 4E). As anticipated, we observed aberrant translation patterns of genes during the IVM process of prepubertal goat oocytes compared to adult goat oocytes in vivo maturation (Figure 4F,G). Two hundred and sixty‐nine genes (red dotted box in Figure 4F) displayed up‐regulation in translatome during adult goat oocytes in vivo maturation but failed to do so in prepubertal goats. These genes were involved in crucial processes such as chromosome segregation (*CENPE, CENPF, CENPN, CENPW, SGO2*), regulation of translation (*CONT7, RBM8A, IREB2, TYMS, EIF2A, PUM2*), protein folding (*LMAN1, GRPEL1, CCDC47, CANX, HSPA4L*) and post‐translational protein acetylation (*ESCO1* and *ESCO2*) (Figure 4F,H). Conversely, 606 genes (red dotted box in Figure 4G) exhibited down‐regulation in translatome during adult goat oocytes in vivo maturation but not in prepubertal goats. These genes were associated with intracellular protein transport, methylation, and metabolic processes including sterol metabolic, glycogen metabolic and fructose metabolic (Figure 4G,I). Our results indicated that a substantial number of genes exhibited translational anomalies with enriched functions related to cytoplasmic maturation and chromosome segregation during IVM of prepubertal goat oocytes.

### 3.6 The Gene Translational Activity of Prepubertal Goat Oocytes Differs From That of Adult Goat

**FIGURE 5:**
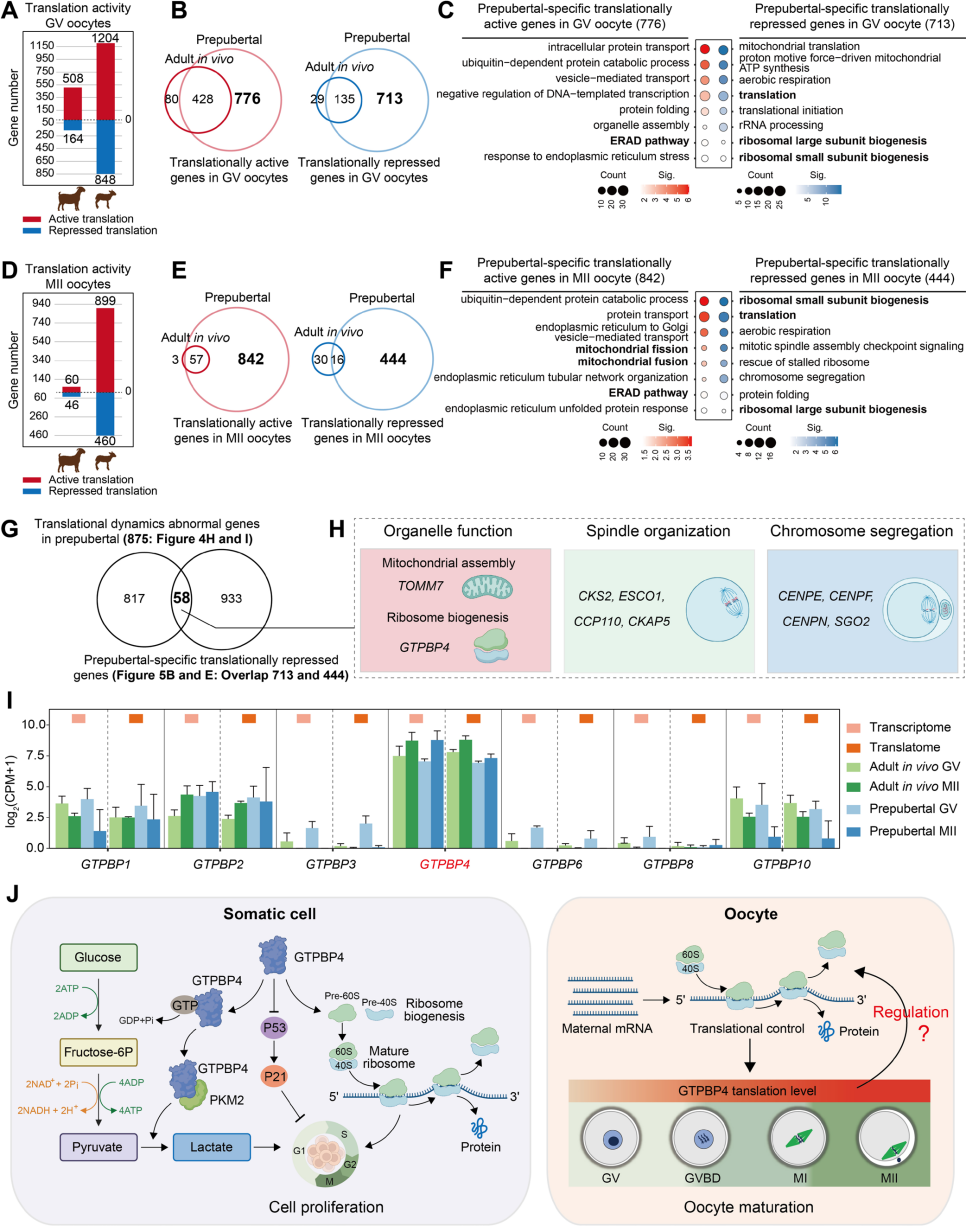
Translational activity of prepubertal goat GV and MII oocytes. (A) Graph showing the number of translationally active genes (red) and translationally repressed genes (blue) in GV oocytes from adult and prepubertal goat. (B) Veen plot shows the overlap of translationally active genes (red) and translationally repressed genes (blue) between adult and prepubertal goat GV oocytes. (C) Representative GO BP terms enrichment of prepubertal‐specific translationally active genes (red) and translationally repressed genes (blue) in GV oocytes. (D) Graph showing the number of translationally active genes (red) and translationally repressed genes (blue) in MII oocytes from adult and prepubertal goat. (E) Veen plot shows the overlap of translationally active genes (red) and translationally repressed genes (blue) between adult and prepubertal goat MII oocytes. (F) Representative GO BP terms enrichment of prepubertal‐specific translationally active genes (red) and translationally repressed genes (blue) in MII oocytes. (G) Veen plot shows the overlap of translational dynamics abnormal genes in prepubertal goat oocytes and prepubertal‐specific translationally repressed genes. (H) The overlapped genes of regulating organelle function, spindle organisation and chromosome segregation showed in Figure 5G. (I) The expression results of GTPBP protein family at transcriptional and translational level during goat oocyte maturation. (J) The biological function of GTPBP4 in somatic cells and its potential role in oocytes.

The regulation of gene expression shifts from transcriptional control to translational regulation during oocyte maturation [32]. Therefore, our objective was to comprehend the translational activity of adult and prepubertal goat GV and MII oocytes. As expected, we observed a substantial number of actively translated genes as well as a significant proportion of translationally suppressed genes, particularly in MII oocytes from prepubertal goats (Figure S8A–C). Further analysis revealed that 776 genes exhibited translational activity specifically in prepubertal goat oocytes but not in adult goats. These genes were primarily involved in intracellular protein transport, protein folding, organelle assembly and the ERAD pathway (Figure 5A–C). Additionally, 713 genes were found to be translationally suppressed exclusively in prepubertal goat oocytes compared to adult goats; these genes were associated with translation regulation, aerobic respiration, rRNA processing and ribosomal biogenesis (Figure 5A–C). A similar pattern was observed for prepubertal goat MII oocytes, where 842 genes displayed translational activity only in these cells but not in adults; these genes were implicated in protein transport, endoplasmic reticulum unfolded protein response, mitochondrial fission and fusion and the ERAD pathway (Figure 5D–F). Furthermore, we identified 444 translationally suppressed genes specific to prepubertal goat oocytes when compared to adults; these genes play roles related to translation regulation mechanisms, aerobic respiration processes, rRNA processing events, chromosome segregation and ribosomal biogenesis (Figure 5D–F). Overall findings indicated the presence of a substantial number of genes associated with abnormal translational activity involved in cytoplasmic processes (Figure S8D). Therefore, we further investigated the cytoplasmic characteristics using transmission electron microscopy. In comparison to MII oocytes from adult goats, prepubertal goat oocytes predominantly exhibited severely shrunken mitochondria, lacking mitochondrial cristae and clustered distribution in the cytoplasm. The endoplasmic reticulum showed swelling, rupture, and ribosome shedding (Figure S9B). Conversely, adult goat oocytes displayed mitochondria with normal morphology characterised by clearly visible mitochondrial cristae and uniformly distributed throughout the cytoplasm. The integrity of the endoplasmic reticulum appeared better (Figure S9A). The functional status of organelles serves as a crucial indicator for the cytoplasmic maturation of oocytes. In prepubertal goat oocytes, the impairment of mitochondria and the endoplasmic reticulum might induce abnormalities in energy metabolism, Ca^2+^ homeostasis, redox homeostasis and protein synthesis within the cytoplasm. These observations suggested that the low developmental potential of prepubertal goat oocytes may also be attributed to immature cytoplasm.

Next, we aimed to identify key factors involved in prepubertal goat oocyte maturation. By comparing the prepubertal‐specific translationally repressed genes (Figure 5B,E: Overlap of 713 and 444 genes) and the genes of aberrant translational dynamics during prepubertal goat oocyte maturation (Figure 4H,I), we identified a total of 58 overlapping genes (Figure 5G). Notably, these genes play crucial roles in various aspects of oocyte maturation, including the regulation of organelle function (*GTPBP4, TOMM7*), spindle organisation (*CKS2, CCP110, CKAP5, ESCO1*) and chromosome segregation (*CENPE, CENPF, CENPN, SGO2*) (Figure 5H and Figure S8E). Furthermore, our observations revealed distinct transcriptional and translational patterns within the GTPBP family during goat oocyte maturation; specifically, *GTPBP4* exhibited high expression levels in goat oocytes (Figure 5I). Interestingly, *GTPBP4* was not significantly altered at the translational level during oocyte maturation in prepubertal goats compared to adult goats (Figure 5I). GTPBP4 is an evolutionarily conserved RNA‐binding proteins (RBPs) across mammals from mice to humans [33] and is involved in ribosome assembly [34]. Given that RBPs are major regulators of mRNA translation for supporting oocyte maturation [35], it suggests that GTPBP4 may be involved in regulating goat oocyte maturation (Figure 5J).

### 3.7 GTPBP4 Improves the Developmental Potential of Prepubertal Goat Oocytes by Promoting Cytoplasmic Maturation

**FIGURE 6:**
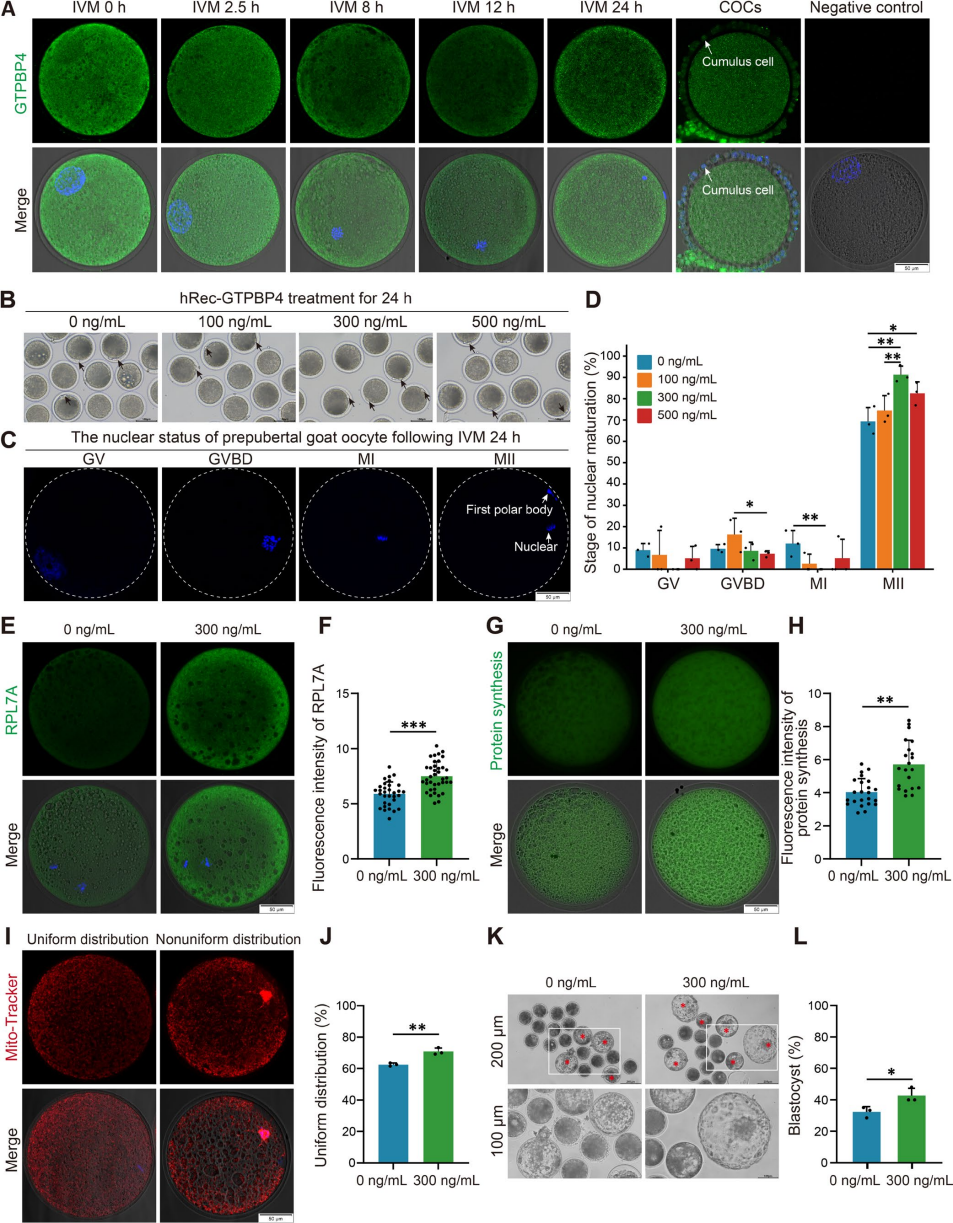
GTPBP4 promotes prepubertal goat oocytes maturation in vitro. (A) Localisation of GTPBP4 during goat oocyte maturation. Green, GTPBP4; blue, DNA; white arrow indicates cumulus cell. Bar = 50 μm. (B) Prepubertal goat oocytes after 24 h of treatment with different concentrations of hRec‐GTPBP4. Black arrow indicates first polar body. Bar = 100 μm. (C) Nuclear conformation were used to define the cell cycle of prepubertal goat oocytes. Bar = 50 μm. (D) After 24 h maturation, the percentage of different meiotic stages of prepubertal goat oocytes in the 0, 100, 300 and 500 ng/mL hRec‐GTPBP4 group. The data were displayed as mean ± SD of three independent experiments. 0 ng/mL, *n* = 53 oocytes; 100 ng/mL, *n* = 55 oocytes; 300 ng/mL *n* = 51 oocytes; and 500 ng/mL *n* = 55 oocytes. *, *p* < 0.05, **, *p* < 0.01. (E) Immunofluorescence staining results of RPL7A in prepubertal goat MII oocytes. Green, RPL7A; blue, DNA. Bar = 50 μm. (F) The fluorescence intensity of RPL7A treated with of 0 ng/mL (*n* = 32 oocytes) and 300 ng/mL (*n* = 38 oocytes) hRec‐GTPBP4. The data were displayed as mean ± SD of three independent experiments. ***, *p* < 0.001. (G) Immunofluorescence staining results of protein synthesis in prepubertal goat MII oocytes. Green, protein synthesis. Bar = 50 μm. (H) The fluorescence intensity of protein synthesis treated with of 0 ng/mL (*n* = 23 oocytes) and 300 ng/mL (*n* = 21 oocytes) hRec‐GTPBP4. The data were displayed as mean ± SD of three independent experiments. **, *p* < 0.01. (I) Mitochondrial distribution pattern in prepubertal goat MII oocytes. Red, mitochondria; blue, DNA. Bar = 50 μm. (J) The uniform distribution ratios of mitochondria treated with 0 ng/mL (*n* = 32 oocytes) and 300 ng/mL (*n* = 38 oocytes) hRec‐GTPBP4. The data were displayed as mean ± SD of three independent experiments. **, *p* < 0.01. (K) Representative images of IVF blastocysts derived from oocytes cultured IVM medium containing 0 and 300 ng/mL hRec‐GTPBP4. The red asterisk indicates blastocyst. Bar = 200 μm, 100 μm. (L) The blastocyst percentages derived from 0 ng/mL (*n* = 64 oocytes) and 300 ng/mL (*n* = 70 oocytes) hRec‐GTPBP4. The data were displayed as mean ± SD of three independent experiments. *, *p* < 0.05. The blastocyst rate were computed on the number of cleavage embryos.

During goat oocyte maturation, GTPBP4 was localised in the cytoplasm of oocytes (Figure 6A). To validate the role of GTPBP4 in enhancing the developmental potential of prepubertal goat oocytes, we investigated the effects of supplementing various concentrations of hRec‐GTPBP4 in IVM medium (Figure 6B). Addition of 300 ng/mL hRec‐GTPBP4 during prepubertal goat GV oocyte maturation resulted in a significant increase in the rate of first polar body extrusion (91.36% ± 3.98% vs. 69.37% ± 6.53%; *p* < 0.01), thereby promoting the cell cycle progression and reducing blockage at the GV phase (0.00% ± 0.00% vs. 9.00% ± 3.06%; *p* = 0.051), GVBD stage (8.64% ± 3.98% vs. 9.62% ± 1.94%; *p* = 0.713) and MI stage (0.00% ± 0.00% vs. 12.02% ± 6.15%; *p* < 0.05) (Figure 6C,D). Moreover, it significantly upregulated RPL7A expression [36], a ribosomal marker (7.49% ± 1.33% vs. 5.89% ± 1.09%; *p* < 0.001) (Figure 6E,F), as well as global protein levels in prepubertal goat MII oocytes (5.71% ± 1.46% vs. 4.03% ± 0.83%; *p* < 0.01) (Figure 6G,H), along with an increased proportion of evenly distributed mitochondria within these cells (70.85% ± 2.18% vs. 62.56% ± 1.05%; *p* < 0.01) (Figure 6I,J). Furthermore, supplementation with hRec‐GTPBP4 at a concentration of 300 ng/mL had a positive impact on IVF blastocyst developmental outcomes compared to the control condition (42.67% ± 4.62% vs. 32.30% ± 3.33%; *p* < 0.05) (Figure 6K,L and Table S1). Collectively, these findings suggested that the addition of hRec‐GTPBP4 can significantly enhance organelle maturation and mRNA translation, thereby improving the developmental potentiality of prepubertal goat oocytes.

## 4 Discussion

In this study, more than 70% of homologous genes shared by goats, humans, and mice exhibited distinct expression patterns in oocytes (Figure 1E), highlighting the species‐specific characteristics of the transcriptome and translatome in these cells [24, 37]. These observed differences likely reflect the evolutionary adaptation to species‐specific requirements [37]. Consequently, relying solely on functional studies conducted using model organisms during oocyte maturation may not accurately elucidate gene function in both human and livestock contexts. These findings underscore the importance of our investigation into goat oocyte maturation through a comprehensive analysis of transcriptome and translatome datasets.

Fully grown oocytes exhibited transcriptional silence while translational regulation of maternal mRNA assumes a dominant role during maturation [38]. In this study, we observed that the gene expression in goat GV oocytes was primarily regulated at the translational level, whereas transcription and translation of genes were closely coupled in MII oocytes. In GV oocytes, it was critical that certain proteins be synthesised in sufficient quantities to initiate oocyte maturation [39], such as *CDC25A*, which exhibits higher translation activity and ensures G2/M transition of goat GV oocytes [40, 41, 42]. Previous studies in mice and humans have shown that high translational expression during oocyte maturation was controlled by specific regulatory elements bound by RBPs to the 3′‐UTR of the genes [24, 43, 44]. In our experiment, ELAVL1 and RALY were found to bind to the 3′ UTR of transcripts with higher translational activity in goat GV oocytes and may potentially regulate mRNA translation during oocyte maturation [45, 46]. However, global translation becomes less active in MII oocytes due to its essential role in maintaining MII arrest and effectively preventing parthenogenetic activation prior to fertilisation [39, 47, 48]. This highlights the crucial importance of mRNA translation regulation during oocyte meiosis.

In this experiment, it was observed that there was a global sequential transition of gene expression regulation from cytoplasm to chromosome segregation during adult and prepubertal oocyte maturation, and this transition of gene regulation was conserved across species [24, 37, 49]. However, translatome data revealed aberrant translational dynamics during prepubertal goat oocyte maturation compared to adults. For instance, genes associated with spindle assembly and chromosome segregation such as *ESCO1* [50], *ESCO2* [51], *CKS2* [52], *SGO2* [53, 54, 55], *CCP110* [56], *CKAP5* [56] and CENP family (*CENPE, CENPF, CENPN, CENPW*) [57, 58, 59, 60] were up‐regulated in the translatome during adult goat oocyte maturation but showed insufficient translation in prepubertal goats. This inadequate translation may result in compromised nuclear maturation. Furthermore, the downregulation of steroid metabolism‐related genes translation during adult goat oocyte maturation was crucial for MII arrest; however continuous translation of these genes occurs in prepubertal goat oocytes leading to spontaneous activation and escape from MII arrest even without fertilisation [61, 62]. Consequently, the low first polar body extrusion rate observed in prepubertal goat oocytes may be attributed to abnormal translation dynamics of genes during the maturation process.

The developmental competence of oocytes from prepubertal females was widely acknowledged to be lower than that of oocytes from adult females, primarily due to immature cytoplasm [18]. In this study, integrated transcriptome and translatome profiling revealed aberrant translation activity of numerous genes in prepubertal goat oocytes compared to adult goat oocytes, implicating their involvement in cytoplasmic maturation. The endoplasmic reticulum is an organelle crucial for protein secretion, modification and folding and clearance of misfolded proteins [63, 64]. Our data demonstrate a significant quantity of translationally active genes involved in the ERAD pathway in prepubertal goat oocytes. Moreover, ultrastructural analysis revealed swelling, rupture and ribosome shedding within the ER. These observations indicated the presence of a large number of misfolded proteins in prepubertal goat oocytes, which require activation of the ERAD pathway for clearance [65, 66]. Similarly, reduced translation activity associated with *TOMM7* involved in mitochondrial assembly may contribute to prominent mitochondrial shrinkage characterised by indiscernible mitochondrial ridge and cluster distribution within the cytoplasm of prepubertal goat oocytes [67, 68]. Furthermore, the RBPs are essential for regulating mRNA translation in oocytes [69]. As an RBP, the diminished translational activity of *GTPBP4* may result in mRNA translation disorder within prepubertal goat oocytes [33]. Moreover, the ribosome is an organelle for translating the genetic information carried by mRNA into protein [70]. The lower translation activity of *GTPBP4* involved in ribosome assembly in prepubertal goat oocytes may lead to abnormal ribosome biogenesis and thus insufficient protein synthesis [71, 72]. Collectively, these findings suggest that abnormal gene translation activity could impair cytoplasmic maturation processes in prepubertal goat oocytes.

During the goat oocyte maturation process, GTPBP4 exhibits a homogeneous distribution throughout the cytoplasm (Figure 6A). However, in somatic cells, the GTPBP4 protein localises to the nucleus [34] and plays a crucial role in ribosome synthesis and maturation within the 60s subunit [71], thereby regulating diverse biological processes including intracellular protein synthesis, signal transduction pathways, cell growth and differentiation [73, 74]. These findings suggested that GTPBP4 displays cell‐specific localisation and performs different functions in oocytes and somatic cells. Furthermore, supplementation of hRec‐GTPBP4 to prepubertal oocytes IVM medium not only enhances ribosome content and protein synthesis but also improves mitochondrial distribution ratio uniformly while increasing the rate of the first polar body extrusion. Collectively, these results indicated that GTPBP4 has significant potential for enhanced both cytoplasmic maturation and nuclear maturation of prepubertal goat oocytes.

## 5 Conclusion

In summary, we have elucidated the dynamic transitions of the transcriptome and translatome during adult and prepubertal goat oocyte maturation. Notably, a sequential shift in gene expression regulation from cytoplasmic processes to chromosome segregation was observed throughout this developmental process. The dysregulation of genes involved in cytoplasmic translation and organelle organisation may contribute to the suboptimal quality of IVM in prepubertal goat oocytes. Importantly, we identified GTPBP4 as a key regulator of goat oocyte maturation—a member of the GTPBP family protein—which has potential implications for enhanced prepubertal goat oocyte maturation by promoting cell cycle progression, increasing ribosome content, ensuring more evenly distributed mitochondria and facilitating mRNA translation. Overall, our findings not only shed light on the mechanism underlying mammalian oocyte maturation but also provide valuable insights for improving juvenile female oocytes IVM.

## Author Contributions

Jianpeng Qin, Yaozong Wei and Ao Ning contributed equally to this work. They were responsible for the data acquisition and analysis. Jianpeng Qin and Keren Cheng were writing the original draft. All the authors performed the goat superovulation, oocytes collection and IVC experiments. Wenqi Hu provided the protocol for the T&T‐seq. Pengcheng Wan, Guozhi Yu, Qiuxia Liang, Liuhong Shen, Lin Zhang and Xiang Chen provided advice for the design of the study. Keren Cheng, Li Meng and Guangbin Zhou were responsible for the conception, design and supervision of the study.

## Conflicts of Interest

The authors declare no conflicts of interest.

## Supporting information


**Figure S1.** Schematic diagram depicting major procedures of goat oocytes collection. (A) Procedure for collection of oocytes from adult goat (in vivo). (B) Procedure for collection of oocytes from adult goat (in vitro). (C) Procedure for collection of oocytes from prepubertal goat.

**Figure S2.** Profiling the genes expression of goat oocytes by T&T‐seq. (A) The gene expression of goat oocytes in transcriptional and translational levels. (B and C) Scatter plots comparing transcriptome (B) and translatome (C) between adult goat (in vivo) and human GV oocytes. (D and E) Scatter plots comparing transcriptome (D) and translatome (E) between adult goat (in vivo) and human MII oocytes. (F and G) Scatter plots comparing transcriptome (F) and translatome (G) between adult goat (in vivo) and mouse GV oocytes. (H and I) Scatter plots comparing transcriptome (H) and translatome (I) between adult goat (in vivo) and mouse MII oocytes.

**Figure S3.** Representative GO BP terms of up‐regulated and down‐regulated genes during adult goat oocyte in vivo maturation. A and B respectively represent down‐regulated and up‐regulated genes.

**Figure S4.** The difference of transcriptome and translatome between adult goat oocytes during in vivo and in vitro maturation. (A) Scatter plots comparing average gene expression values between GV and MII oocytes in transcriptional levels. (B and C) Representative GO BP terms enrichment of up‐regulated (red colour circle) and down‐regulated (blue colour circle) genes showed in Figure S3A, respectively. (D) Scatter plots comparing average gene expression values between GV and MII oocytes in translational levels. (E and F) Representative GO BP terms enrichment of up‐regulated (red colour circle) and down‐regulated (blue colour circle) genes showed in Figure S3D, respectively. (G) Alluvial diagram showing the kinetics of gene expression during maturation of adult goat oocytes in vitro. (H) Representative GO BP terms of different classes of genes showed in Figure S3G, respectively. (I) Veen plot shows the overlap of DEGs identified by transcriptome between in vivo and in vitro. (J) Veen plot shows the overlap of DEGs identified by translatome between in vivo and in vitro.

**Figure S5.** Representative GO BP terms of up‐regulated and down‐regulated genes during adult goat oocyte in vitro maturation. A and B respectively represent down‐regulated and up‐regulated genes.

**Figure S6.** Dynamics of transcriptome and translatome during maturation of prepubertal goat oocytes. (A and B) Representative GO BP terms enrichment of up‐regulated (red colour circle) and down‐regulated (blue colour circle) genes in transcriptome. (C and D) Representative GO BP terms enrichment of up‐regulated (red colour circle) and down‐regulated (blue colour circle) genes in translatome. (E) Alluvial diagram showing the kinetics of gene expression during maturation of prepubertal goat oocytes. (F) Representative GO BP terms of different classes of genes showed in Figure S4E, respectively.

**Figure S7.** Representative GO BP terms of up‐regulated and down‐regulated genes during prepubertal goat oocyte in vitro maturation. A and B respectively represent down‐regulated and up‐regulated genes.

**Figure S8.** Translational activity of prepubertal goat oocytes. (A and B) Scatter plots comparing average gene expression values between transcriptional and translational levels from prepubertal goat GV and MII oocytes, respectively. (C) Pattern of gene expression in prepubertal goat oocytes. (D) The biological process of translational activity abnormal genes in prepubertal GV and MII oocytes. (E) Translational expression levels of the representative genes of adult and prepubertal goat GV and MII oocytes.

**Figure S9.** Observation of goat oocytes ultrastructure. (A) Representative images of adult (in vivo) goat MII oocytes obtained via electron microscopy. Bar = 2 μm, 1 μm, 500 nm. (B) Representative images of prepubertal goat MII oocytes obtained via electron microscopy. Bar = 2 μm, 1 μm, 500 nm. ZP: Zona pellucida, PVS: Perivitelline space, Mit: Mitochondria, Mi: Microvilli, CGs: Cortical granules, LD: Lipid droplets, Va: Vacuoles, ER: Endoplasmic reticulum. The purple arrowhead indicates normal mitochondria, the red arrowhead indicates mitochondrial solidification, the blue arrowhead indicates mitochondrial vacuoles, the green arrowhead indicates normal endoplasmic reticulum, the yellow arrowhead indicate the endoplasmic reticulum that is swollen, ruptured and ribosome shedding.

**Table S1.** The effect of hRec‐GTPBP4 on development of prepubertal goat oocytes after in vitro fertilisation.

Data S1.

Data S2.

Data S3.

Data S4.

## Data Availability

The data that support the findings of this study are openly available in NCBI SRA at https://www.ncbi.nlm.nih.gov/sra/docs/, reference number PRJNA1130873.
