# Supplementary figures and images for: Decoding the Molecular Landscape of Prepubertal Oocyte Maturation: GTPBP4 as a Key Driver of In Vitro Developmental Competence
Source: Cell Prolif. 2025 Feb 28;58(11):e70017. doi: 10.1111/cpr.70017 (PMC12584866; doi:10.1111/cpr.70017)

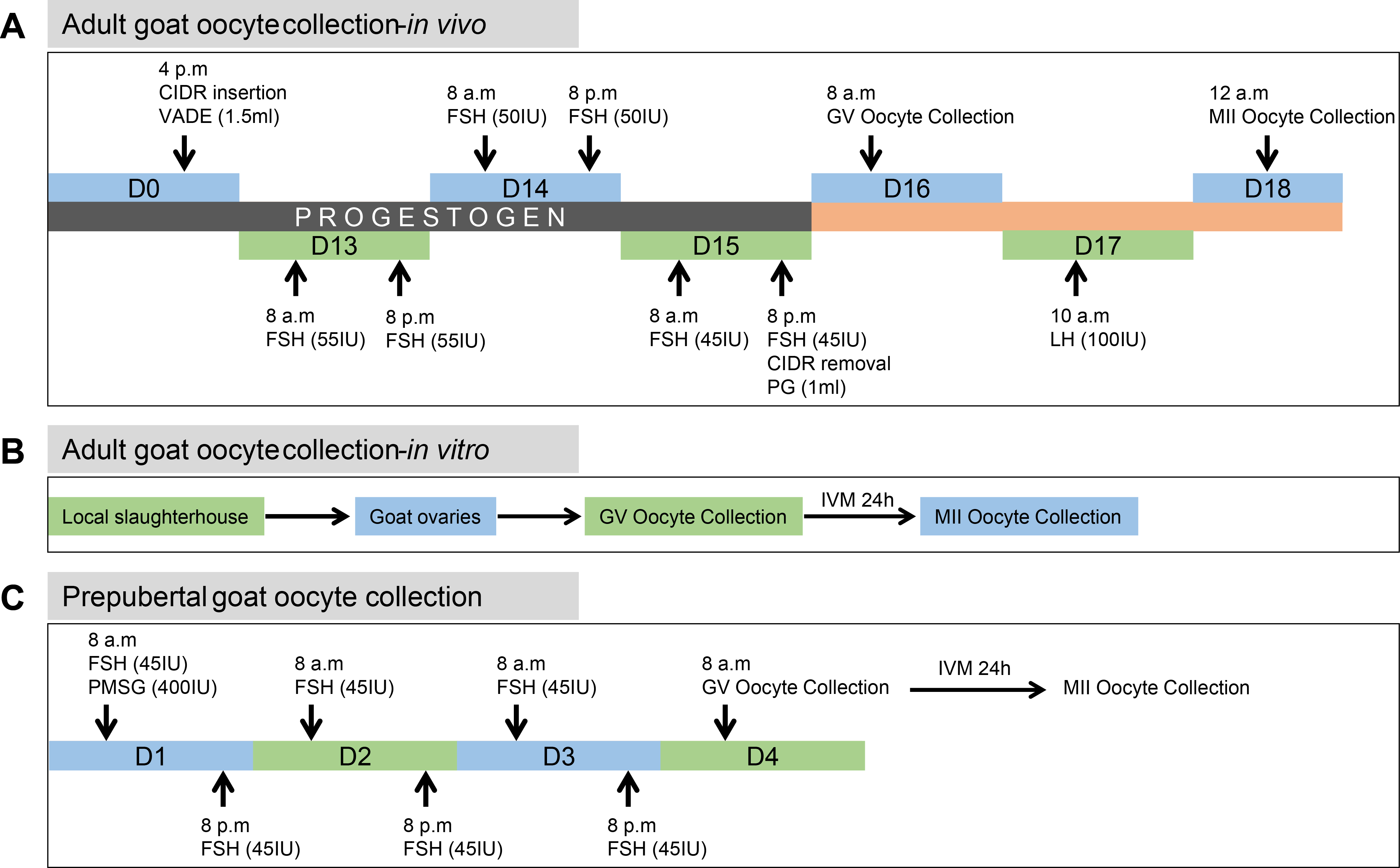

Supplement: Supplementary file 1 — Figure S1. Schematic diagram depicting major procedures of goat oocytes collection. (A) Procedure for collection of oocytes from adult goat (in vivo). (B) Procedure for collection of oocytes from adult goat (in vitro). (C) Procedure for collection of oocytes from prepubertal goat. [file CPR-58-e70017-s011.jpg]

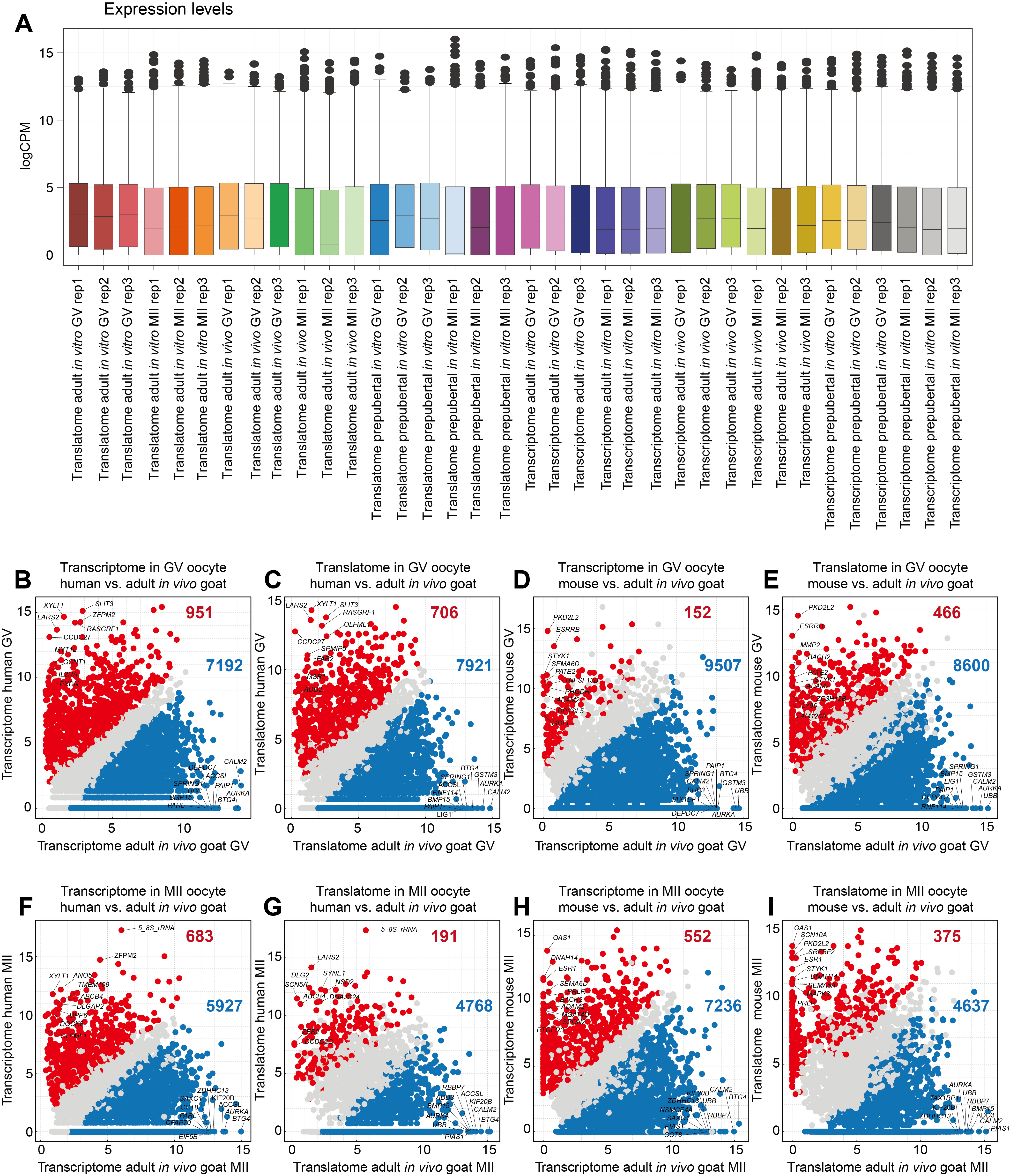

Supplement: Supplementary file 2 — Figure S2. Profiling the genes expression of goat oocytes by T&T‐seq. (A) The gene expression of goat oocytes in transcriptional and translational levels. (B and C) Scatter plots comparing transcriptome (B) and translatome (C) between adult goat (in vivo) and human GV oocytes. (D and E) Scatter plots comparing transcriptome (D) and translatome (E) between adult goat (in vivo) and human MII oocytes. (F and G) Scatter plots comparing transcriptome (F) and translatome (G) between adult goat (in vivo) and mouse GV oocytes. (H and I) Scatter plots comparing transcriptome (H) and translatome (I) between adult goat (in vivo) and mouse MII oocytes. [file CPR-58-e70017-s010.jpg]

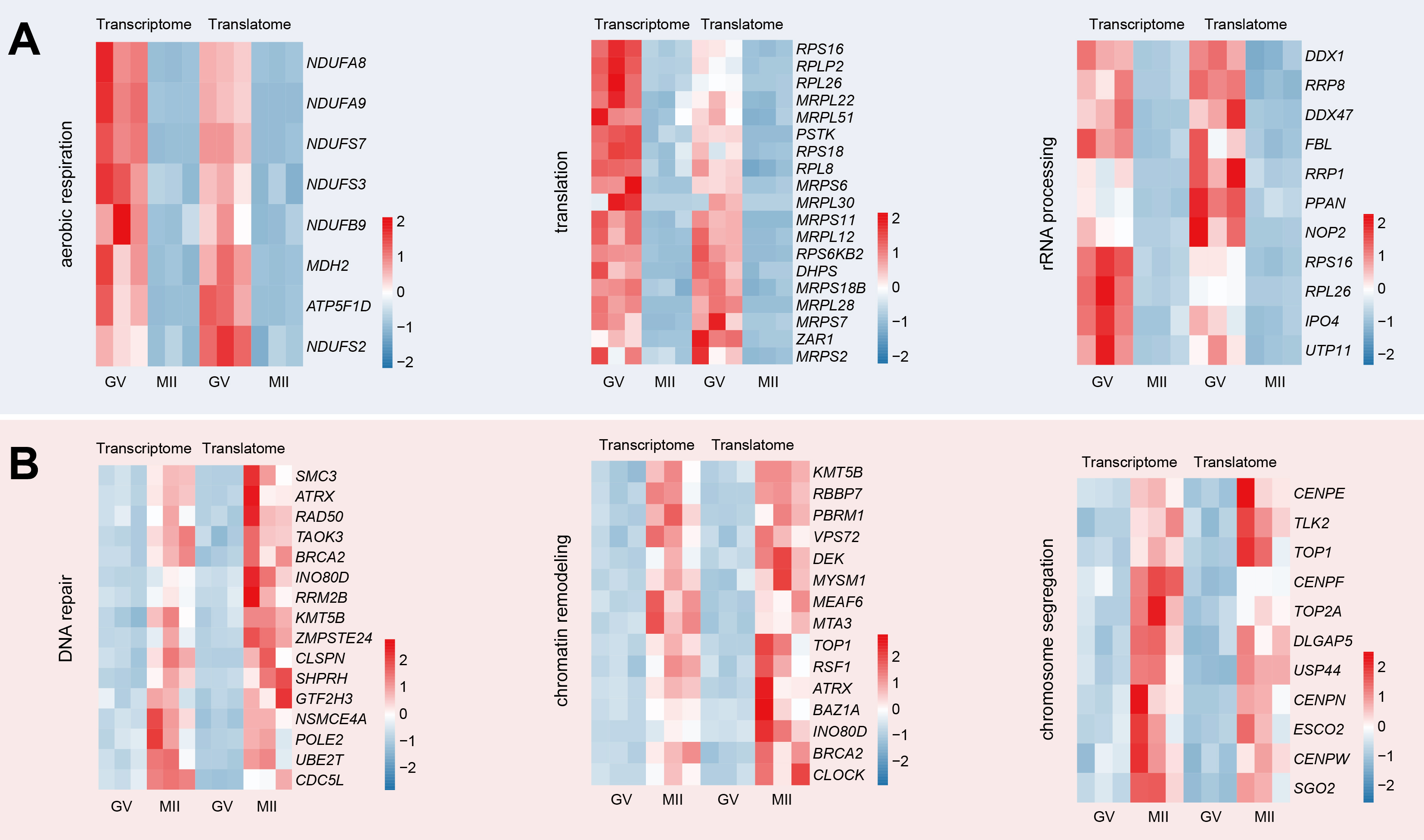

Supplement: Supplementary file 3 — Figure S3. Representative GO BP terms of up‐regulated and down‐regulated genes during adult goat oocyte in vivo maturation. A and B respectively represent down‐regulated and up‐regulated genes. [file CPR-58-e70017-s009.jpg]

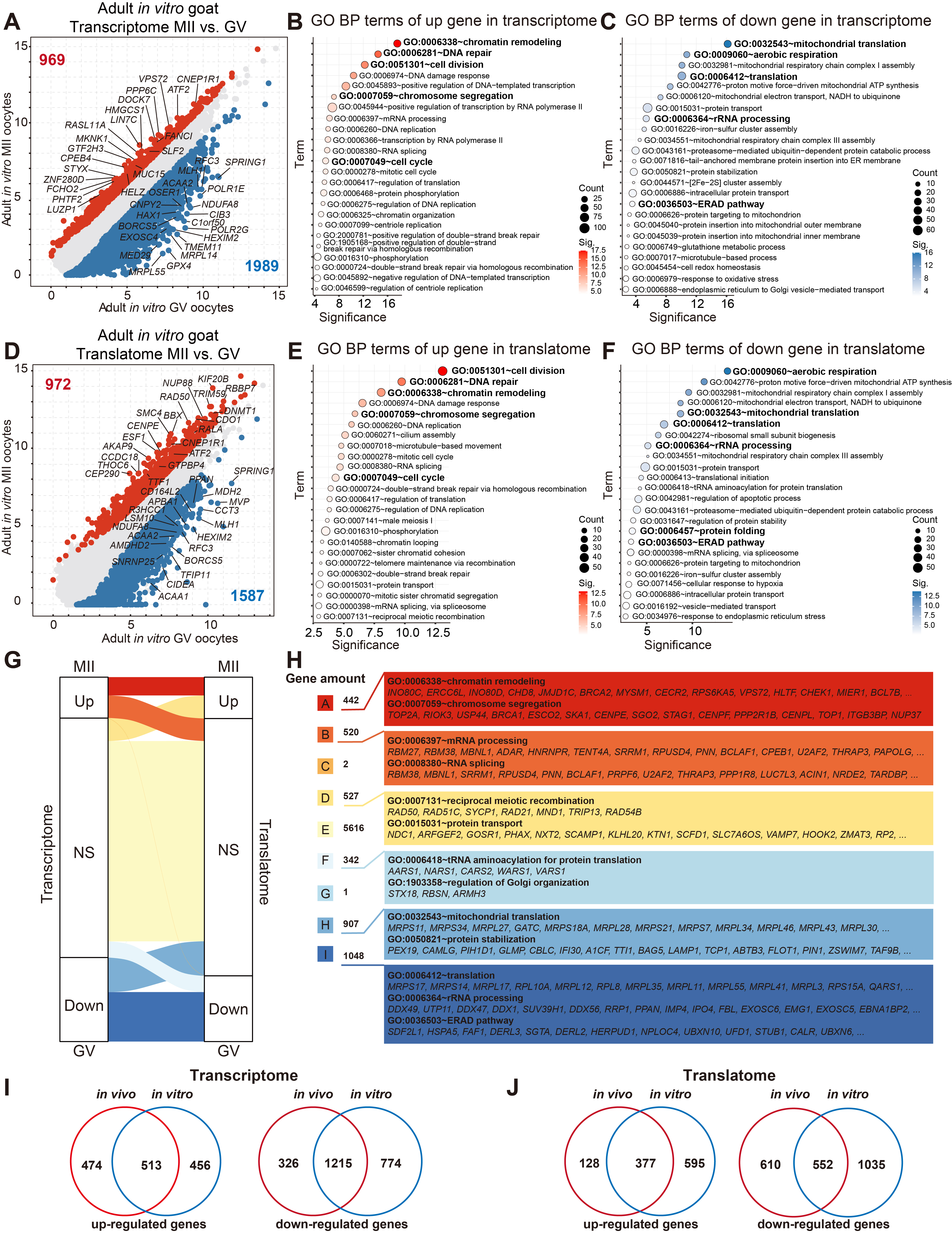

Supplement: Supplementary file 4 — Figure S4. The difference of transcriptome and translatome between adult goat oocytes during in vivo and in vitro maturation. (A) Scatter plots comparing average gene expression values between GV and MII oocytes in transcriptional levels. (B and C) Representative GO BP terms enrichment of up‐regulated (red colour circle) and down‐regulated (blue colour circle) genes showed in Figure S3A, respectively. (D) Scatter plots comparing average gene expression values between GV and MII oocytes in translational levels. (E and F) Representative GO BP terms enrichment of up‐regulated (red colour circle) and down‐regulated (blue colour circle) genes showed in Figure S3D, respectively. (G) Alluvial diagram showing the kinetics of gene expression during maturation of adult goat oocytes in vitro. (H) Representative GO BP terms of different classes of genes showed in Figure S3G, respectively. (I) Veen plot shows the overlap of DEGs identified by transcriptome between in vivo and in vitro. (J) Veen plot shows the overlap of DEGs identified by translatome between in vivo and in vitro. [file CPR-58-e70017-s005.jpg]

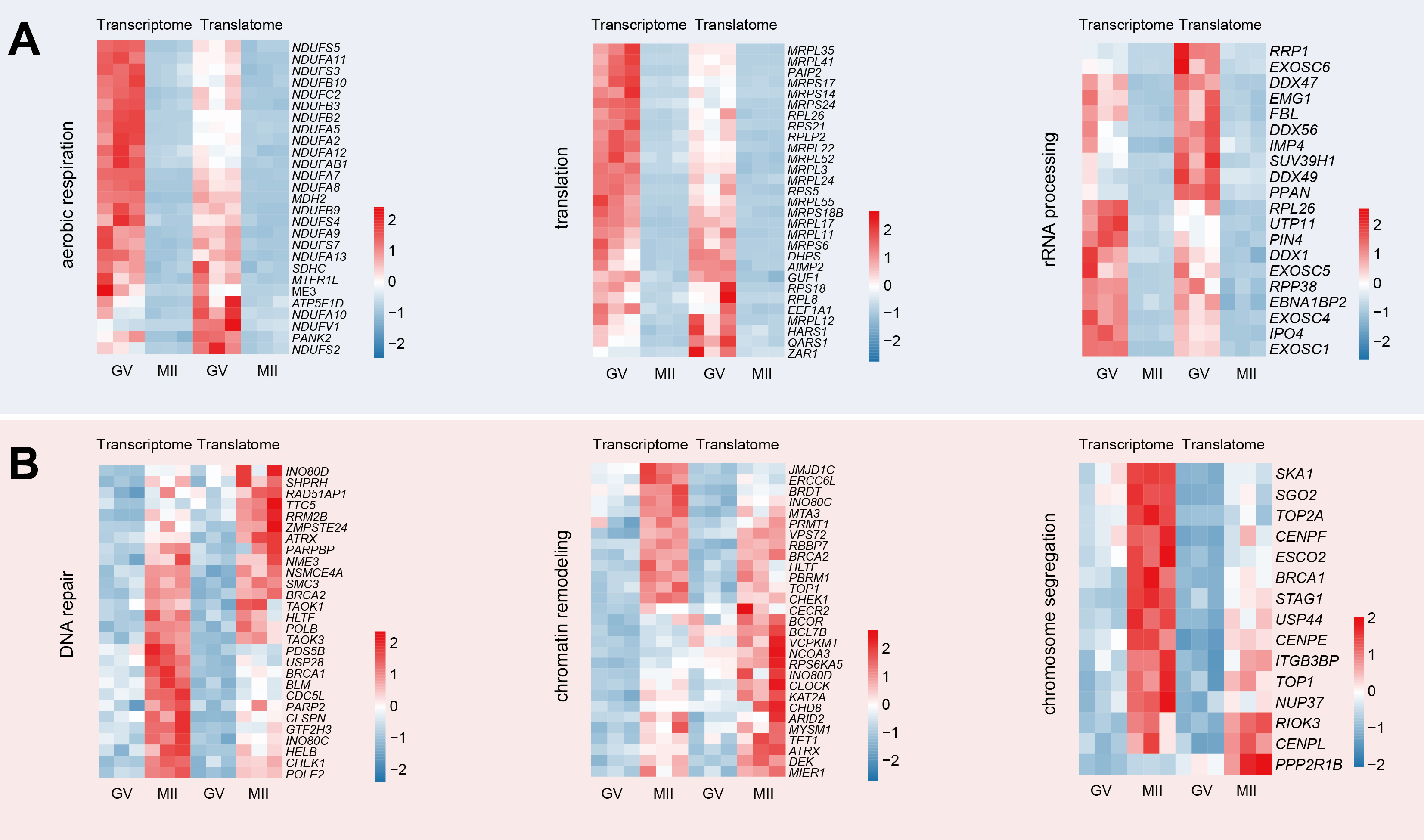

Supplement: Supplementary file 5 — Figure S5. Representative GO BP terms of up‐regulated and down‐regulated genes during adult goat oocyte in vitro maturation. A and B respectively represent down‐regulated and up‐regulated genes. [file CPR-58-e70017-s014.jpg]

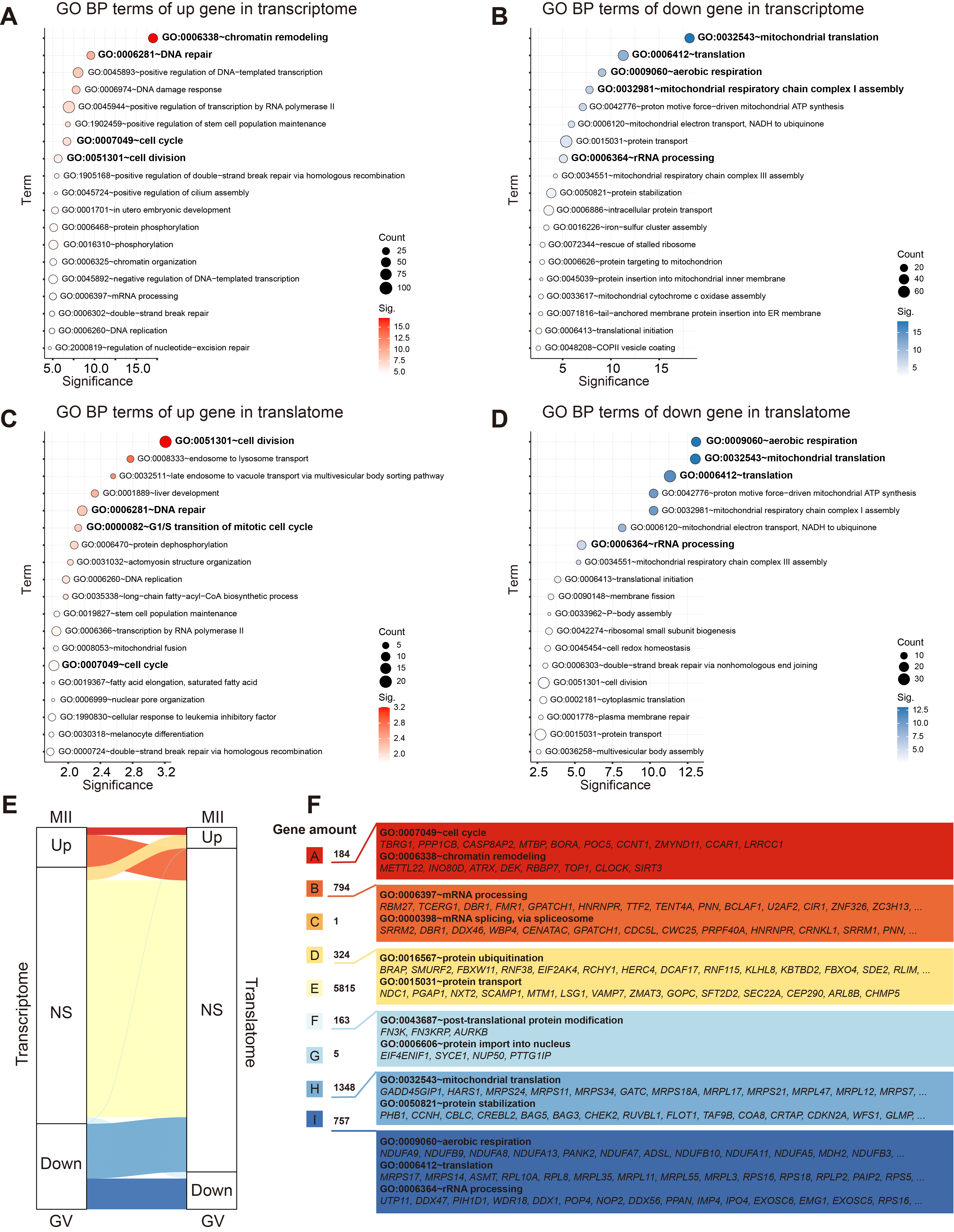

Supplement: Supplementary file 6 — Figure S6. Dynamics of transcriptome and translatome during maturation of prepubertal goat oocytes. (A and B) Representative GO BP terms enrichment of up‐regulated (red colour circle) and down‐regulated (blue colour circle) genes in transcriptome. (C and D) Representative GO BP terms enrichment of up‐regulated (red colour circle) and down‐regulated (blue colour circle) genes in translatome. (E) Alluvial diagram showing the kinetics of gene expression during maturation of prepubertal goat oocytes. (F) Representative GO BP terms of different classes of genes showed in Figure S4E, respectively. [file CPR-58-e70017-s001.jpg]

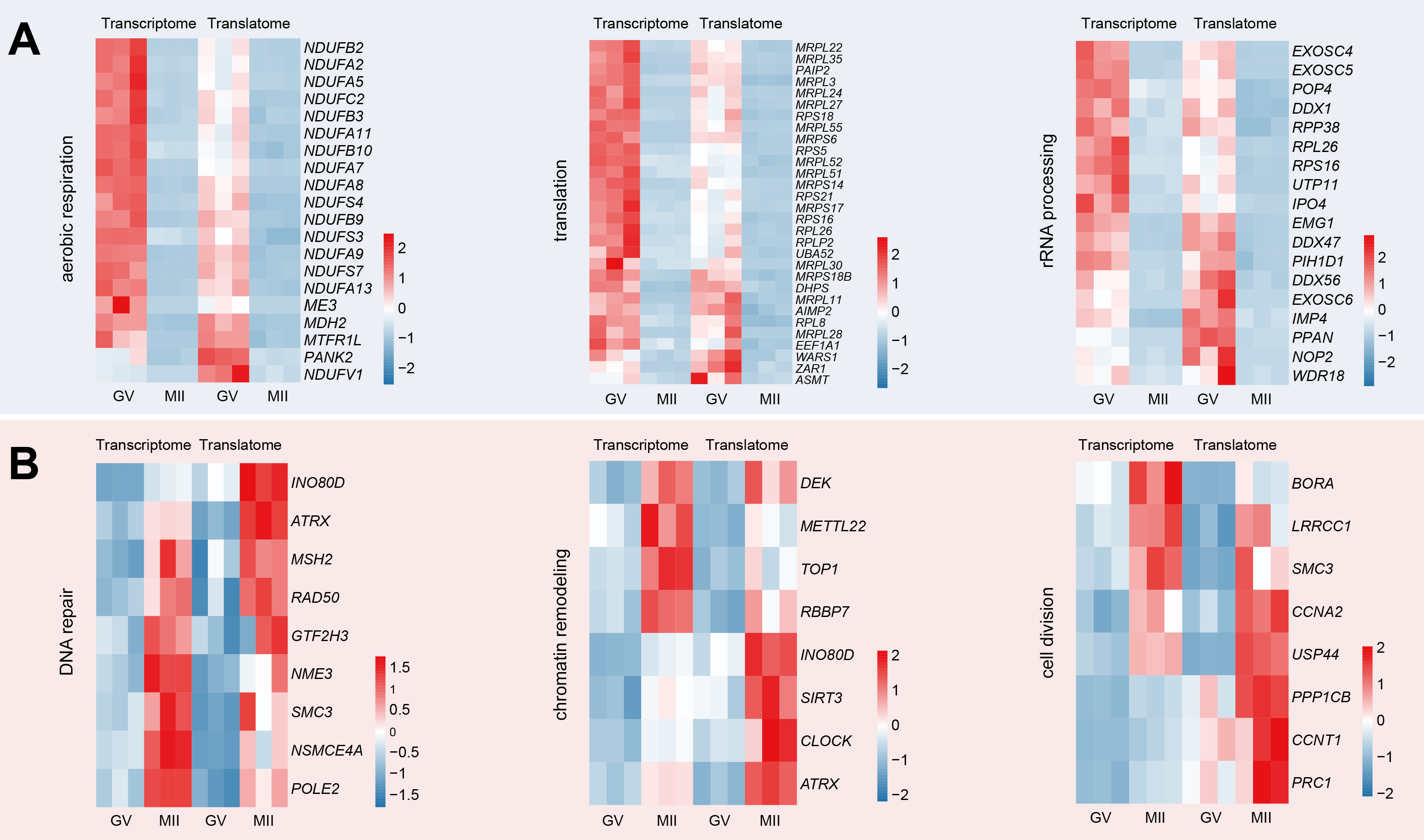

Supplement: Supplementary file 7 — Figure S7. Representative GO BP terms of up‐regulated and down‐regulated genes during prepubertal goat oocyte in vitro maturation. A and B respectively represent down‐regulated and up‐regulated genes. [file CPR-58-e70017-s004.jpg]

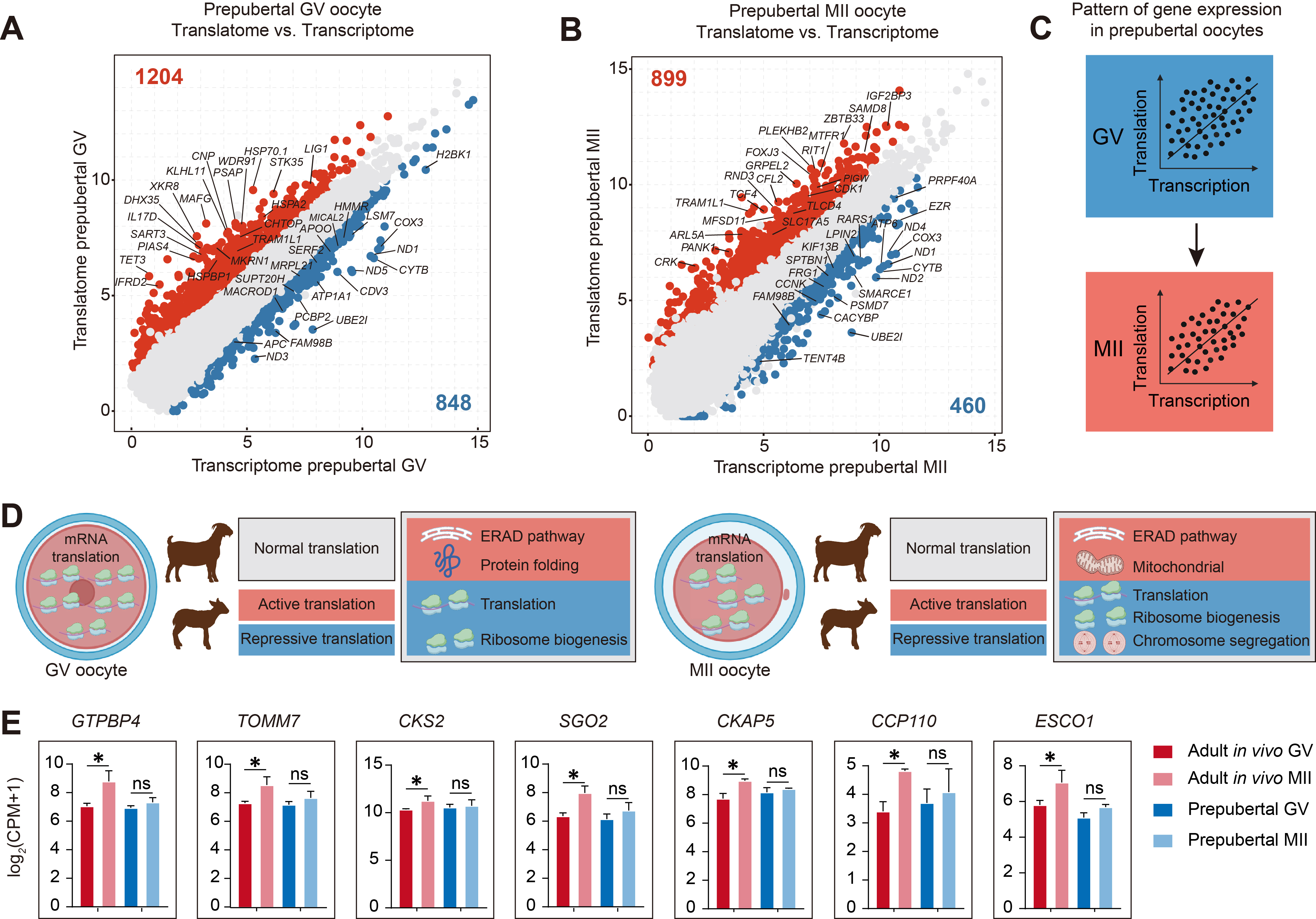

Supplement: Supplementary file 8 — Figure S8. Translational activity of prepubertal goat oocytes. (A and B) Scatter plots comparing average gene expression values between transcriptional and translational levels from prepubertal goat GV and MII oocytes, respectively. (C) Pattern of gene expression in prepubertal goat oocytes. (D) The biological process of translational activity abnormal genes in prepubertal GV and MII oocytes. (E) Translational expression levels of the representative genes of adult and prepubertal goat GV and MII oocytes. [file CPR-58-e70017-s006.jpg]

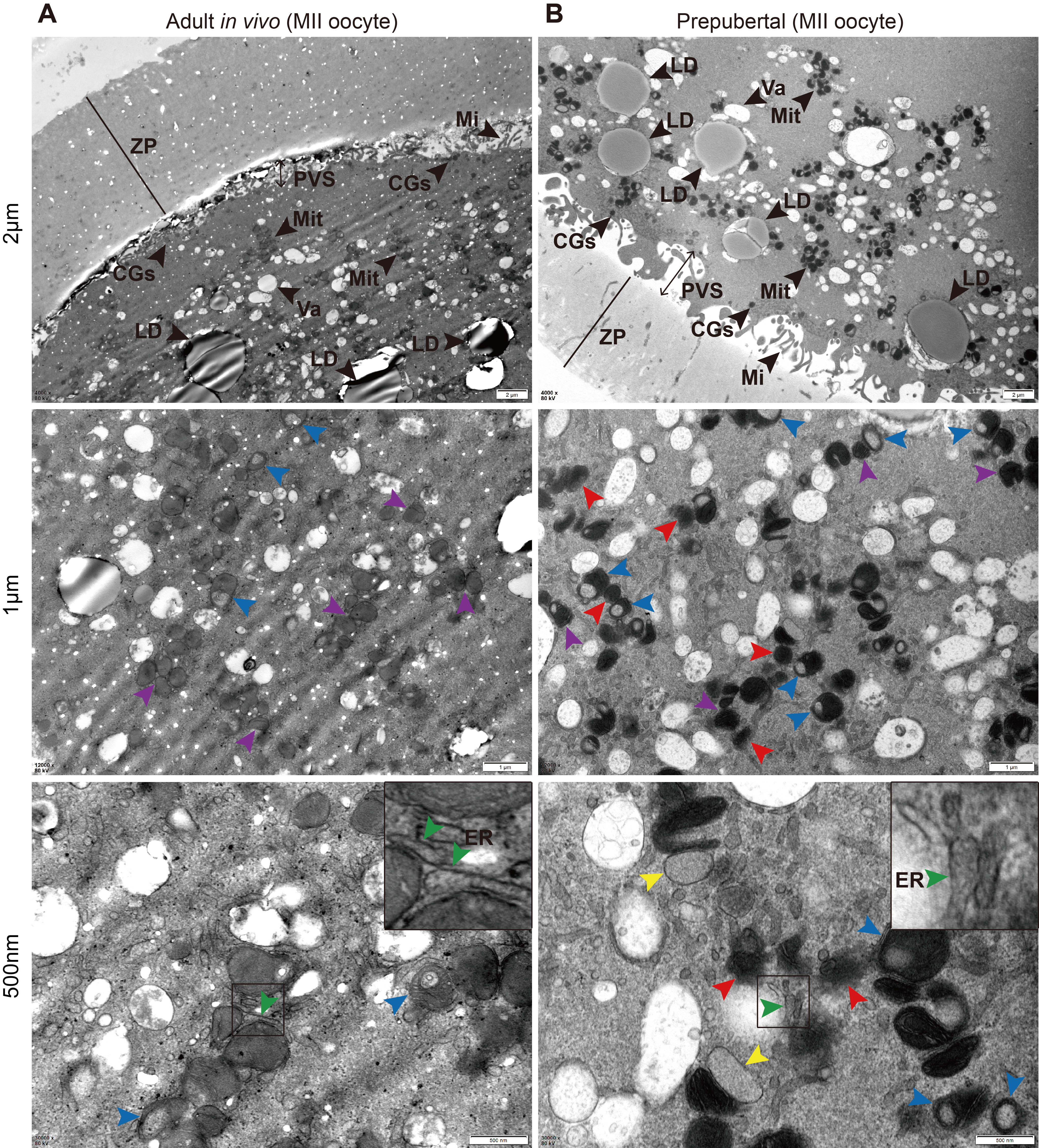

Supplement: Supplementary file 9 — Figure S9. Observation of goat oocytes ultrastructure. (A) Representative images of adult (in vivo) goat MII oocytes obtained via electron microscopy. Bar = 2 μm, 1 μm, 500 nm. (B) Representative images of prepubertal goat MII oocytes obtained via electron microscopy. Bar = 2 μm, 1 μm, 500 nm. ZP: Zona pellucida, PVS: Perivitelline space, Mit: Mitochondria, Mi: Microvilli, CGs: Cortical granules, LD: Lipid droplets, Va: Vacuoles, ER: Endoplasmic reticulum. The purple arrowhead indicates normal mitochondria, the red arrowhead indicates mitochondrial solidification, the blue arrowhead indicates mitochondrial vacuoles, the green arrowhead indicates normal endoplasmic reticulum, the yellow arrowhead indicate the endoplasmic reticulum that is swollen, ruptured and ribosome shedding. [file CPR-58-e70017-s002.jpg]
