# Supplementary material for: Decoding the Molecular Landscape of Prepubertal Oocyte Maturation: GTPBP4 as a Key Driver of In Vitro Developmental Competence
Source: Cell Prolif. 2025 Feb 28;58(11):e70017. doi: 10.1111/cpr.70017 (PMC12584866; doi:10.1111/cpr.70017)
Supplement: Supplementary file 10 — Table S1. The effect of hRec‐GTPBP4 on development of prepubertal goat oocytes after in vitro fertilisation. [file CPR-58-e70017-s012.docx]

Supplementary table 1. The effect of hRec-GTPBP4 on development of prepubertal goat oocytes after *in vitro* fertilization

| Treatment | IVF COCs (n) | Cleavage (n, %) | Blastocysts-1 (n, %) | Blastocysts-2 (n, %) |
| --- | --- | --- | --- | --- |
| 0 ng/mL | 64 | 46 (72.55 ± 3.17) | 15 (23.40 ± 2.08) | 15 (32.30 ± 3.33) |
| 300 ng/mL | 70 | 60 (85.32 ± 3.44)** | 26 (36.51 ± 5.50)* | 26 (42.67 ± 4.62)* |

The numbers of IVF COCs derived from prepubertal goat GV oocytes cultured IVM medium containing 0 ng/mL and 300 ng/mL hRec-GTPBP4. The numbers of cleavage embryos and blastocyst were counted at day 2 and day 7 after fertilization respectively. The rates of cleavage embryo and blastocysts-1 were calculated from the number of IVF COCs, the rates of blastocysts-2 were calculated from the number of cleavage embryos. Data are presented as mean ± SD of three independent experiments. *, *P* < 0.05; **, *P* < 0.01.
